# Novel nano-reinforced chitosan derivatives for adsorption of heavy metal ions from aqueous media

**DOI:** 10.1038/s41598-026-62595-z

**Published:** 2026-07-21

**Authors:** Ahmed M. Abdelmaksoud, Mohamed E. A. Ali, Gasser M. Khairy, Nadia F. Abdel-Aal

**Affiliations:** 1 Chemistry Department, Faculty of Science, Suze Canal University, Ismailia, 41522 Egypt; 2 Hydro Geochemistry Department, Desert Research Center, Egypt Desalination Research Center of Excellence (EDRC), Cairo, 11753 Egypt

**Keywords:** Chitosan-EDTA, Metal-organic frameworks, Adsorption capacity, Heavy metals, Ethylenediamine tetra acetic dianhydride, Chemistry, Environmental sciences, Materials science, Nanoscience and technology

## Abstract

A novel NH_2_-MIL-53(Al)@CS-EDTA hybrid nanocomposite was synthesized by functionalizing chitosan with EDTA and integrating it with an Al-based MOF for heavy metal removal. Structural and morphological characterizations were performed using FT-IR, XRD, SEM-EDX, N2 adsorption-desorption isotherms, point of zero charge (PZC), and thermogravimetric analysis (TGA). Batch experiments showed maximum adsorption capacities of 31.8, 28.1, and 21 mg·g^− 1^ for Cu^2+^, Fe^2+^, and Ni^2+^ at pH 6. The adsorption data were best fitted by the Langmuir isotherm and the pseudo-second-order kinetic model under the investigated experimental conditions. The observed adsorption behavior is proposed to involve coordination and electrostatic interactions between the metal ions and the abundant functional groups (–OH, –NH₂, and –COOH) of the composite. The biopolymer MOF composite’s reusability was also evaluated, and even after five consecutive adsorption-desorption cycles, > 90% efficiency was found, indicating acceptable stability over repeated cycles. The novelty of this study lies in the synergistic integration of biopolymer, chelating agent, and MOF into a single, reusable adsorbent with improved properties.

## Introduction

Water is a fundamental resource for life and development, yet global water security is increasingly threatened by population growth, industrialization, and climate change^1–8^. Among the most hazardous water contaminants are heavy metal ions such as Pb²⁺, Cu²⁺, Ni²⁺, Fe²⁺, and Cd²⁺, which originate from industrial effluents including pharmaceuticals, batteries, tanning, and fertilizers^9^. These ions are non-biodegradable and tend to accumulate in living organisms, causing severe health effects including organ damage, neurological disorders, and even fatalities^10,11^. Therefore, efficient removal of such pollutants—particularly Cu²⁺, Ni²⁺, and Fe²⁺—from water sources has become a critical environmental challenge. Several treatment techniques have been developed to remove heavy metals from water, including chemical precipitation^12^, ion exchange^13,14^, membrane separation, and adsorption. Among these, adsorption has proven to be a highly effective and economically viable method due to its simplicity, low operational cost, and reusability^15,16^.

Chitosan, the second most abundant biopolymer, has gained attention for its chelation capacity due to the presence of multiple amine and hydroxyl groups^17,18^. However, its mechanical weakness and limited adsorption performance have prompted researchers to modify its structure using functional groups. One of the most effective modifications involves grafting with ethylenediaminetetraacetic acid (EDTA), a well-known chelating agent capable of forming stable complexes with heavy metal ions^19–24^. In parallel, metal–organic frameworks (MOFs) such as NH₂-MIL-53(Al) have shown great promise as adsorbents due to their tunable functional groups^25^. NH_2_-MIL-53(Al) is amino-functionalized metal-organic framework (MOF) named after the Materials Institute Lavoisier (MIL), with an aluminum (Al) metal center and 2-amino terephthalic acid (NH2-BDC) as a ligand. It is a type of porous material with the MIL-53(Al) framework, featuring the addition of an amino group (NH_2_) to the organic linker, which provides enhanced reactivity and functionality for various applications, such as sensing, gas separation, and catalysis. The integration of MOFs with modified biopolymers may result in advanced hybrid materials with synergistic properties for enhanced adsorption. MOF-based adsorbents have attracted considerable attention in wastewater treatment because of their exceptionally tunable pore structures, structural diversity, and the possibility of introducing specific functional groups capable of selectively interacting with target contaminants. These characteristics make MOFs promising materials for the removal of heavy metals, dyes, pharmaceuticals, and other emerging pollutants from aqueous media. However, the practical application of pristine MOFs still faces several challenges, including the limited water stability of certain frameworks, difficulty in recovering fine MOF particles after treatment, potential aggregation, regeneration efficiency, synthesis cost, and shows potential for future practical evaluation. Consequently, recent studies have focused on the development of hybrid adsorbents, such as MOF–polymer and MOF–COF composites, to improve structural stability, recyclability, accessibility of adsorption sites, and overall treatment performance under realistic operating condition^26^.

Compared with pristine MOFs, functionalized MOFs and polymer–MOF hybrid materials offer several additional advantages for wastewater treatment applications. Surface functionalization can introduce specific binding groups that enhance selectivity and adsorption affinity toward target pollutants, while polymer incorporation may improve mechanical stability, processability, and recyclability. In particular, biopolymer–MOF hybrids combine the structural tunability of MOFs with the abundant functional groups of natural polymers, resulting in improved adsorption performance and stability under aqueous conditions. Nevertheless, excessive functionalization or polymer loading may partially block MOF pores, reduce accessible surface area, and introduce mass-transfer limitations. Therefore, achieving an appropriate balance between functionality, and structural stability remains a key challenge in the design of high-performance hybrid adsorbents for practical wastewater treatment applications.

Recent research has explored MOF–chitosan composites and EDTA-functionalized adsorbents individually for heavy metal removal. However, most existing materials suffer from limitations such as low mechanical stability, reduced reusability, or selective adsorption of specific ions. To date, limited studies have explored the integration of these three components into a single system—chitosan, EDTA, and NH₂-MIL-53(Al)— forming a reusable nanocomposite. This represents a significant gap in the current literature, especially for materials capable of simultaneously removing multiple metal ions under realistic environmental conditions.

In this study, a novel hybrid of biopolymer MOF EDTA nanocomposite—NH₂-MIL-53(Al)@CS-EDTA—was synthesized and evaluated for its ability to adsorb Cu²⁺, Ni²⁺, and Fe²⁺ ions from aqueous solutions. Adsorption performance was assessed under varying pH, contact time, concentration, and the presence of competitive ions. CS- EDTA is also synthesized, and its performance was compared with that of the hybrid biopolymer MOF-EDTA nanocomposite. The composite’s structural stability and reusability were also examined, supporting its potential as an environmentally friendly solution for industrial wastewater treatment.

## Experimental

### Materials

Chitosan (degree of deacetylation 93%, molecular weight ≈ 161.16 g/mol) was purchased from Oxford Lab Chem (India). EDTA (ethylenediaminetetraacetic acid), sodium hydroxide (NaOH), acetic anhydride, dry diethyl ether, and pyridine were obtained from Spectrochem Specialty Reagents (India). N, N-dimethylformamide (DMF), aluminum chloride hexahydrate (AlCl₃·6 H₂O), 2-amino terephthalic acid (NH₂-BDC), and ethanol (98%) were supplied by Sigma-Aldrich (Germany). All chemicals were of analytical grade and used without further purification. Deionized water was used for all solution preparations. Stock solutions of Cu²⁺, Ni²⁺, and Fe²⁺ (1000 mg L^−1^) were prepared by dissolving appropriate amounts of CuCl₂·2 H₂O, NiCl₂, and FeSO₄·7 H₂O in deionized water. Working solutions ranging from 1 to 200 mg L^−1^ were obtained via serial dilution. The pH was adjusted using 0.1 M NaOH or 0.1 M HCl.

### Synthesis of EDTA di-anhydride

A definite amount of EDTA (100 g) was suspended in 160 mL of pyridine. After adding 140 g of acetic anhydride, the mixture was stirred for 24 h at 65 °C. The product was then filtered, washed with diethyl ether and acetic anhydride respectively for at least three times, and allowed to dry for 24 h^29^.

### Synthesis of NH₂-MIL-53(Al)

NH₂-MIL-53(Al) nanocrystals were synthesized based on the method of Yu et al.^30^ with minor modifications. The structure is shown in Scheme 2^31^. In a solvent mixture of DMF and distilled water (1:5 v/v, 30 mL total), 108.6 mg of AlCl₃·6 H₂O and 75 mg of NH₂-BDC were dissolved under stirring. The solution was transferred into a 50 mL Teflon-lined stainless-steel autoclave and heated at 120 °C for 24 h. After cooling to room temperature, the resulting white precipitate was collected by centrifugation, washed three times with DMF and ethanol, and dried under vacuum at 60 °C for 24 h.

### Synthesis of chitosan-EDTA

**Scheme 1:**
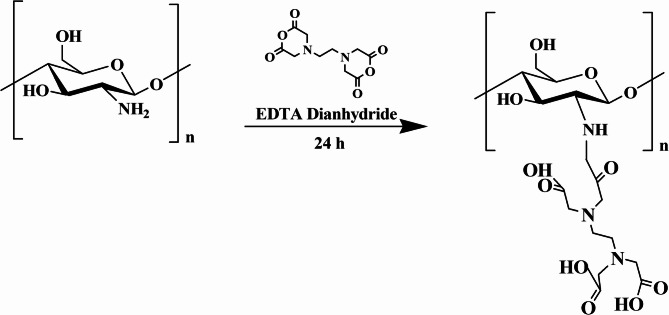
Synthesis scheme of CS-EDTA.

Approximately 3 g of chitosan was dissolved in 180 mL of 6% (v/v) acetic acid and stirred overnight. 12 g of previously prepared EDTA dianhydride (dissolved in 180 mL methanol) was added and stirred overnight. The product was filtered, soaked in ethanol for 2 h, then in NaOH solution (0.1 M) for another 2 h to neutralize unreacted acids. The final product, denoted as CS-EDTA, was washed repeatedly with distilled water until neutral pH, and dried in a desiccator. The reaction pathway is illustrated in Scheme 1^32^.

### Synthesis of NH₂-MIL-53(Al)@chitosan-EDTA nanocomposite

**Scheme 2:**
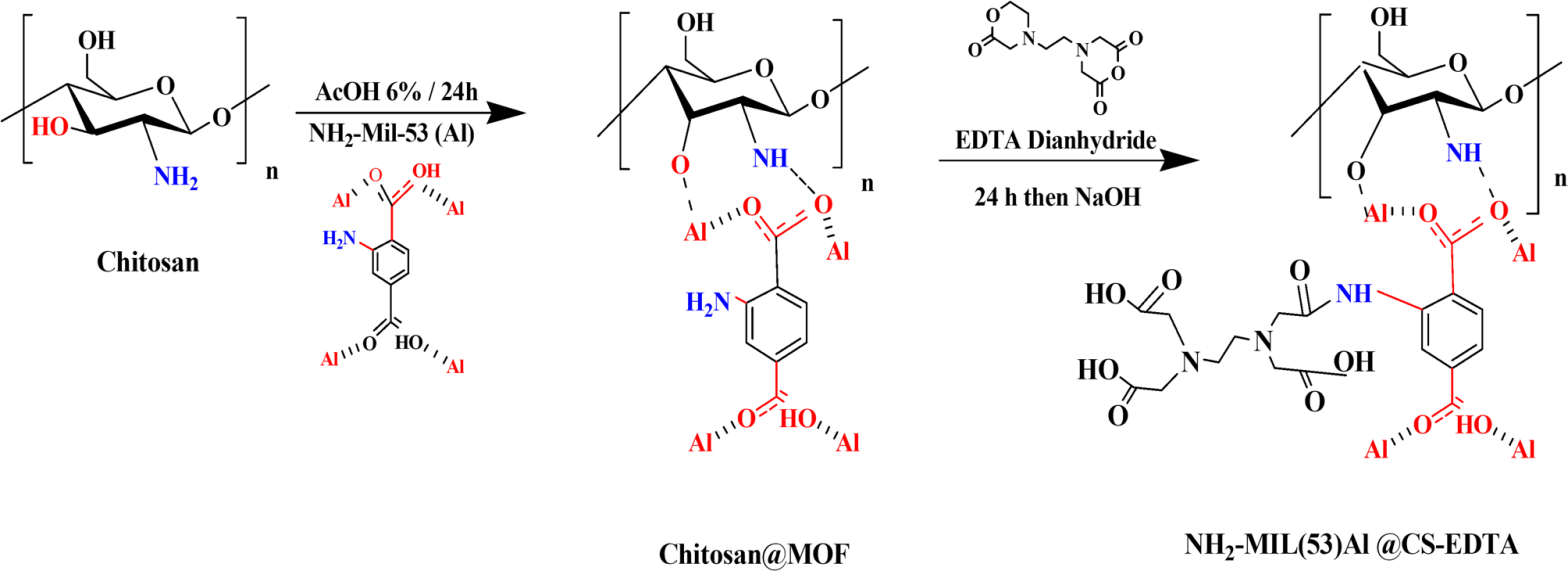
Synthesis scheme of NH2-MIL-53 (Al) @ CS-EDTA.

Approximately 3 g of chitosan was dissolved in 180 mL of 6% (v/v) acetic acid and stirred overnight. Separately, 0.06 g of NH₂-MIL-53(Al) was dispersed in 30 mL methanol and 20 mL water via ultrasonication, then added dropwise to the chitosan solution and stirred for 90 min. To this mixture, 12 g of previously prepared EDTA dianhydride (dissolved in 180 mL methanol) was added and stirred overnight. The product was filtered, soaked in ethanol for 2 h, then in NaOH solution (0.1 M) for another 2 h to neutralize unreacted acids. The final product, denoted as NH₂-MIL-53(Al)@CS-EDTA, was washed repeatedly with distilled water until neutral pH, and dried in a desiccator. The reaction pathway is illustrated in Scheme 2.

.

### Characterization techniques

FT-IR spectra were recorded using a Nicolet Nexus 679 (USA) to confirm functional group modifications and chemical bonding. Crystallographic structures were analyzed using a PANalytical X’Pert PRO diffractometer with CuKα radiation (λ = 1.5406 Å), operating at 30 mA and 40 kV within a 2θ range of 4–90°. Surface morphology and elemental composition were observed via SEM (QUANTA FEG 250) equipped with energy-dispersive X-ray spectroscopy (EDX). BET surface area and pore properties were analyzed using a Micromeritics ASAP 2020 at 77 K. Thermal stability was evaluated using a TGA analyzer (e.g., Shimadzu TGA-50) from room temperature to 800 °C under nitrogen. Point of Zero Charge (PZC) was determined by pH drift method using 0.01 M NaCl solution as background electrolyte.

### Batch adsorption experiment

Batch adsorption experiments were conducted to evaluate the adsorption performance of the synthesized NH₂-MIL-53(Al)@CS-EDTA composite for the removal of Cu²⁺, Ni²⁺, and Fe²⁺ ions from aqueous solutions. All adsorption experiments were independently performed in triplicate using 100 mL Erlenmeyer flasks containing 20 mL of metal ion solution, agitated on a mechanical shaker at 150 rpm and maintained at room temperature (298 ± 1 K), unless otherwise stated. The reported experimental results represent the mean values of three independent measurements. Standard deviations are presented as error bars in the figures showing direct experimental observations and, where applicable, as ± values in the corresponding data tables.

#### Preparation of metal ion solutions

Stock solutions (1000 mg L^−1^) of Cu²⁺, Ni²⁺, and Fe²⁺ were prepared by dissolving 2.6832 g of CuCl₂·2 H₂O, 4.0499 g of NiCl₂, and 4.8775 g of FeSO₄·7 H₂O in 1 L of deionized water, respectively. Working solutions of desired concentrations (20–100 mg L^−1^) were obtained by serial dilution of the stock solutions using deionized water.

#### Effect of adsorbent dosage

To determine the optimum adsorbent dose, varying amounts of NH₂-MIL-53(Al)@CS-EDTA (0.5, 1.0, 1.5, 2.0, 2.5, and 3.5 g L^−1^) were added to 20 mL of 100 mg L^−1^ metal ion solution. The suspensions were stirred for 1 h, after which the solutions were filtered, and the residual ion concentrations were analyzed. The dosage experiment was conducted as a screening study using a fixed contact time of 1 h to evaluate the relative influence of adsorbent dosage and to identify a suitable operational dosage for subsequent adsorption experiments.

#### Effect of contact time

Kinetic studies were carried out by adding 2.5 g L^−1^ (equivalent to 0.05 g in 20 mL solution) of the adsorbent to the metal ion solution (100 mg L^−1^) at pH 6. The contact time was varied (30, 60, 120, 160, 240, and 1440 min). At each time interval, the mixture was filtered immediately, and the supernatant was analyzed to determine the concentration of remaining metal ions.

#### Effect of pH

The effect of pH on metal ion adsorption was investigated in the pH range of 2–12. The pH was adjusted using 0.1 M NaOH or 0.1 M HCl. A constant adsorbent dose (2.5 g L^−1^), metal ion concentration (100 mg L^−1^), and contact time (1 h) were used. The mixtures were agitated, filtered, and analyzed for residual metal content.

#### Effect of initial metal ion concentration

Initial concentrations of Cu²⁺, Ni²⁺, and Fe²⁺ ranging from 20 to 100 mg L^−1^ were studied under constant conditions: pH 6, adsorbent dose of 2.5 g L^−1^, and contact time of (1 h). The adsorption capacity was evaluated to determine the influence of concentration gradients on adsorption performance.

#### Effect of competitive ions

To examine the selectivity of the adsorbent in the presence of competing ions, binary and ternary systems of Cu²⁺, Ni²⁺, and Fe²⁺ were prepared with equal concentrations (20–100 mg L^−1^). The same batch protocol was followed to evaluate changes in adsorption behavior.

After each adsorption experiment, the mixtures were filtered through 0.22 μm syringe filters, and the residual dissolved metal concentrations were determined by atomic absorption spectroscopy (AAS). For iron adsorption experiments, the reported values correspond to the residual dissolved iron concentration measured by AAS.

For iron adsorption experiments, the residual dissolved iron concentration was determined as total iron by AAS. Since flame AAS does not differentiate between Fe²⁺ and Fe³⁺ species, the oxidation state of iron during the adsorption process was not monitored.

Adsorption capacity (q, mg/g) and removal efficiency (R, %) were calculated using the following equations:

1 $$\:q=\frac{\left(Ci-Cf\right)V}{m}$$

2 $$\:R\%=\frac{\left(Ci-Cf\right)}{Ci}\times\:100$$

Where, Ci and Cf are the initial concentration and the final metal ion concentration. (mg L^−1^), V is the volume of the solution (L), and m is the mass of adsorbent (g).

#### Regeneration of NH₂-MIL-53(Al)@CS-EDTA

A regeneration study of nanocomposite was conducted through multiple adsorption–desorption cycles. Approximately 0.05 g of the adsorbent was added to 20 mL of a 100 mgL^−1^ aqueous solution of Cu²⁺, Fe²⁺, or Ni²⁺ at pH 6 and room temperature. The mixtures were agitated at 150 rpm for 1 h. After separation by filtration, the metal-loaded adsorbent was desorbed using 20 mL of 2 M HNO₃ for 10 min, followed by thorough washing with deionized water to remove residual acid. This adsorption–desorption cycle was repeated for five consecutive runs. The regeneration efficiency (RE%) was calculated using the following equation:

3 $$\:\%RE=\frac{{q}_{r}}{{q}_{o}}\times\:100$$

where q_r_ is the adsorption capacity after regeneration and q_o_ is the initial capacity before regeneration. The adsorption capacity was determined after each cycle to evaluate the efficiency and sustainability of the regeneration process.

## Results and discussion

### Characterization techniques

#### FT-IR spectroscopy

**Fig. 1:**
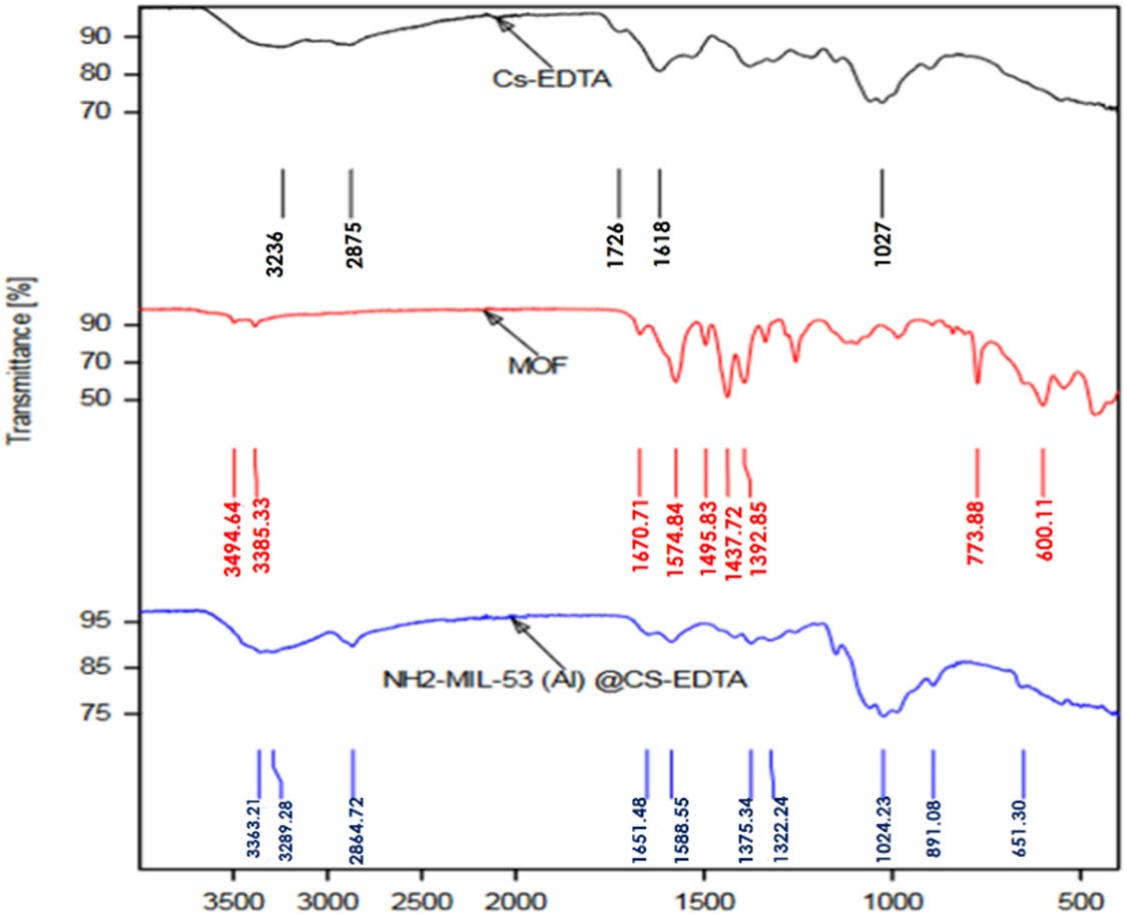
FT-IR spectra of CS-EDTA, NH_2_-MIL-53 (Al), and NH_2_-MIL-53 (Al) @CS-EDTA.

The NH₂-MIL-53(Al)@CS-EDTA nanocomposite was successfully synthesized through a solution-mixing method, as detailed in the experimental section. The interaction among chitosan (CS), ethylenediaminetetraacetic acid (EDTA), and the metal–organic framework (MOF) was primarily governed by the polycationic nature of chitosan in acidic media, which facilitates the protonation of its amino and hydroxyl groups. This protonation promotes strong electrostatic interactions and hydrogen bonding with the functional groups of both EDTA and the MOF. Such interactions are expected to contribute to a homogeneous co-dispersion of the components at the molecular level, while also enhancing the composite’s mechanical integrity and overall stability. The FT-IR spectra presented in Fig. 1 provide compelling evidence for the successful integration of the three components. In the spectrum of CS-EDTA, characteristic absorption bands were observed at 3236 cm^−1^ and 2875 cm^−1^, corresponding to the O–H and C–H stretching vibrations, respectively. The band at 1378 cm^−1^ can be assigned to O–H bending vibrations, which are typically associated with the carboxylic groups and moisture retained in the structure. These peaks confirm the presence of chitosan functionalized with EDTA. In the case of the pure NH₂-MIL-53(Al) spectrum, prominent peaks in the range of 3500–3300 cm^−1^ were attributed to N–H stretching vibrations, indicating the presence of amino functional groups derived from the NH₂-BDC ligand. Additionally, C = O stretching vibrations appeared between 1600 and 1400 cm^−1^, while the bands between 400 and 1000 cm^−1^ were assigned to Al–O stretching vibrations, characteristic of the MOF structure. The FT-IR spectrum of the final nanocomposite, NH₂-MIL-53(Al)@CS-EDTA, revealed new significant peaks at approximately 1024 cm^−1^, 891 cm^−1^, and 651 cm^−1^, which are attributed to Al–O vibrational modes, thus suggesting the formation of the composite and the successful incorporation of the MOF. Notably, strong absorption bands at 3363 cm^−1^ and 3289 cm^−1^, corresponding to the symmetric and asymmetric stretching vibrations of the NH₂ groups, remained prominent in the composite spectrum. The persistence of these functional groups indicates that the structural integrity of the MOF was preserved during the composite formation, and that no undesirable coordination or degradation occurred between the aluminum sites and the amine groups. These spectral observations collectively suggest that NH₂-MIL-53(Al) was successfully embedded within the CS-EDTA matrix without compromising its structural or functional properties, thereby validating the synthetic approach and supporting its potential application in heavy metal ion adsorption.

####  Scanning electron microscopy (SEM) and energy-dispersive X-ray spectroscopy (EDX)

**Fig. 2:**
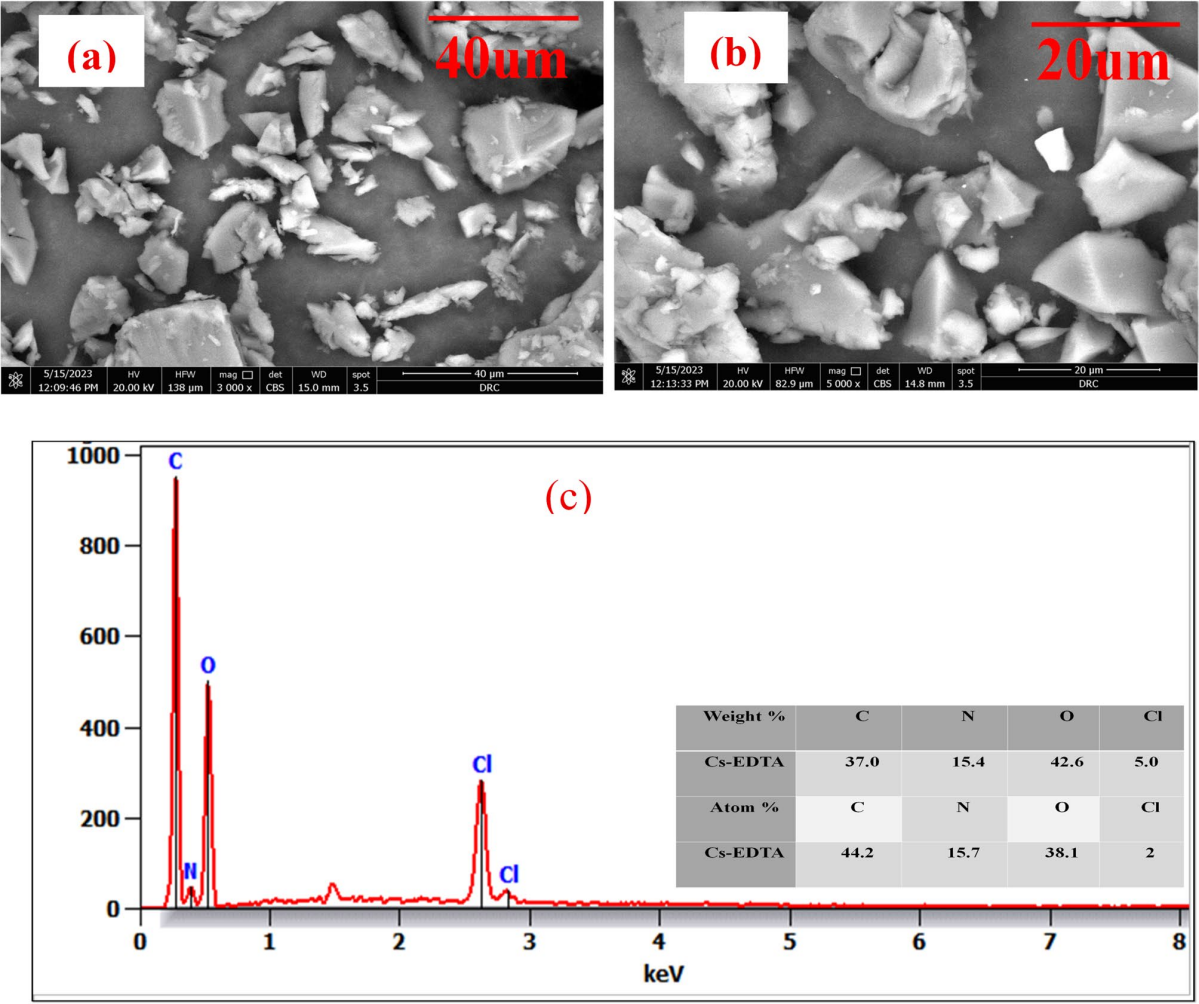
(**a**) SEM image of CS-EDTA with a. low magnification, (**b**) with high magnification and (**c**) EDX analysis of CS-EDTA.

**Fig. 3:**
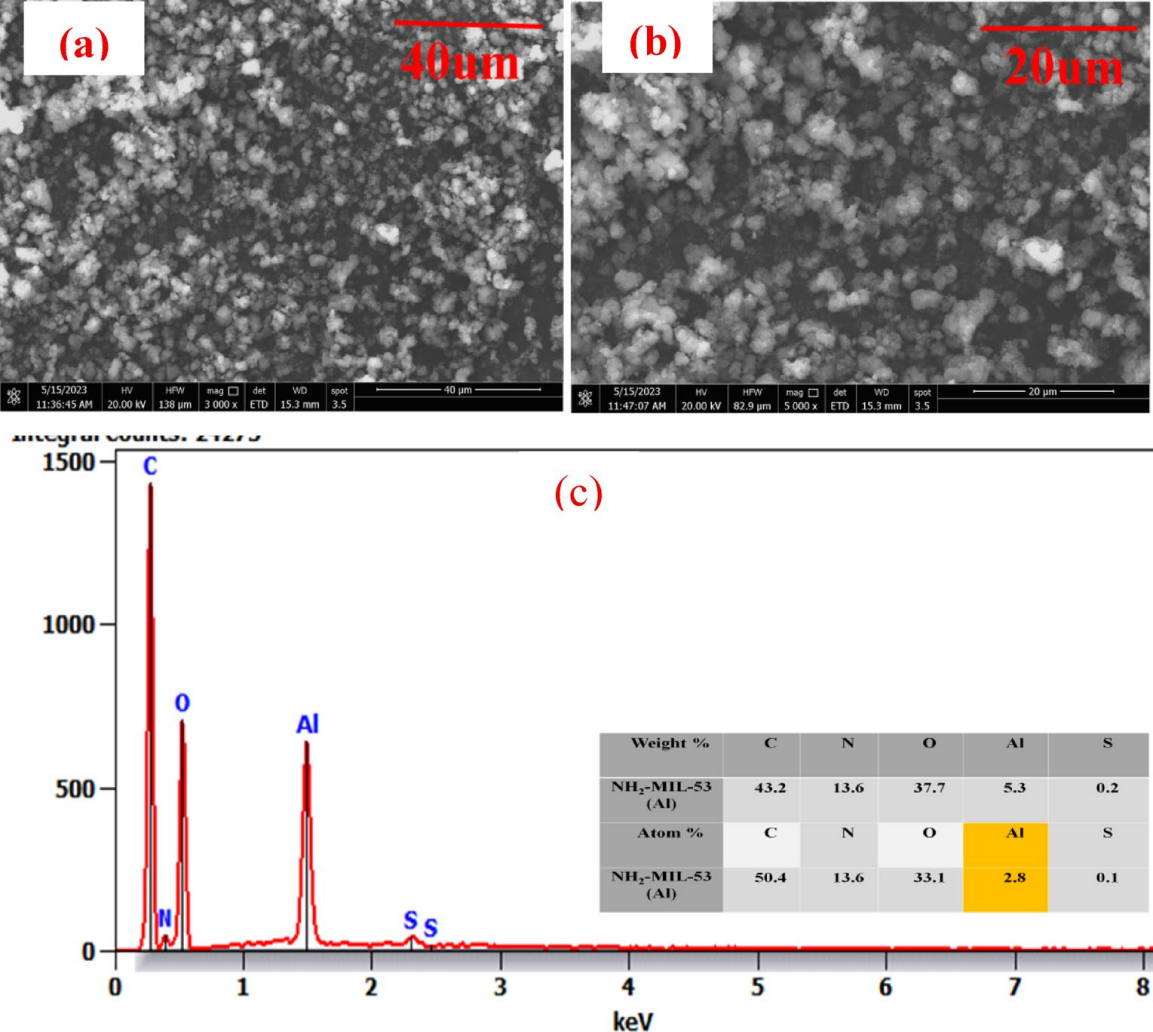
SEM image of NH_2_-MIL-53 (Al) with (**a**) low magnification, (**b**) high magnification and (**c**) EDX analysis of NH_2_-MIL-53 (Al).

**Fig. 4:**
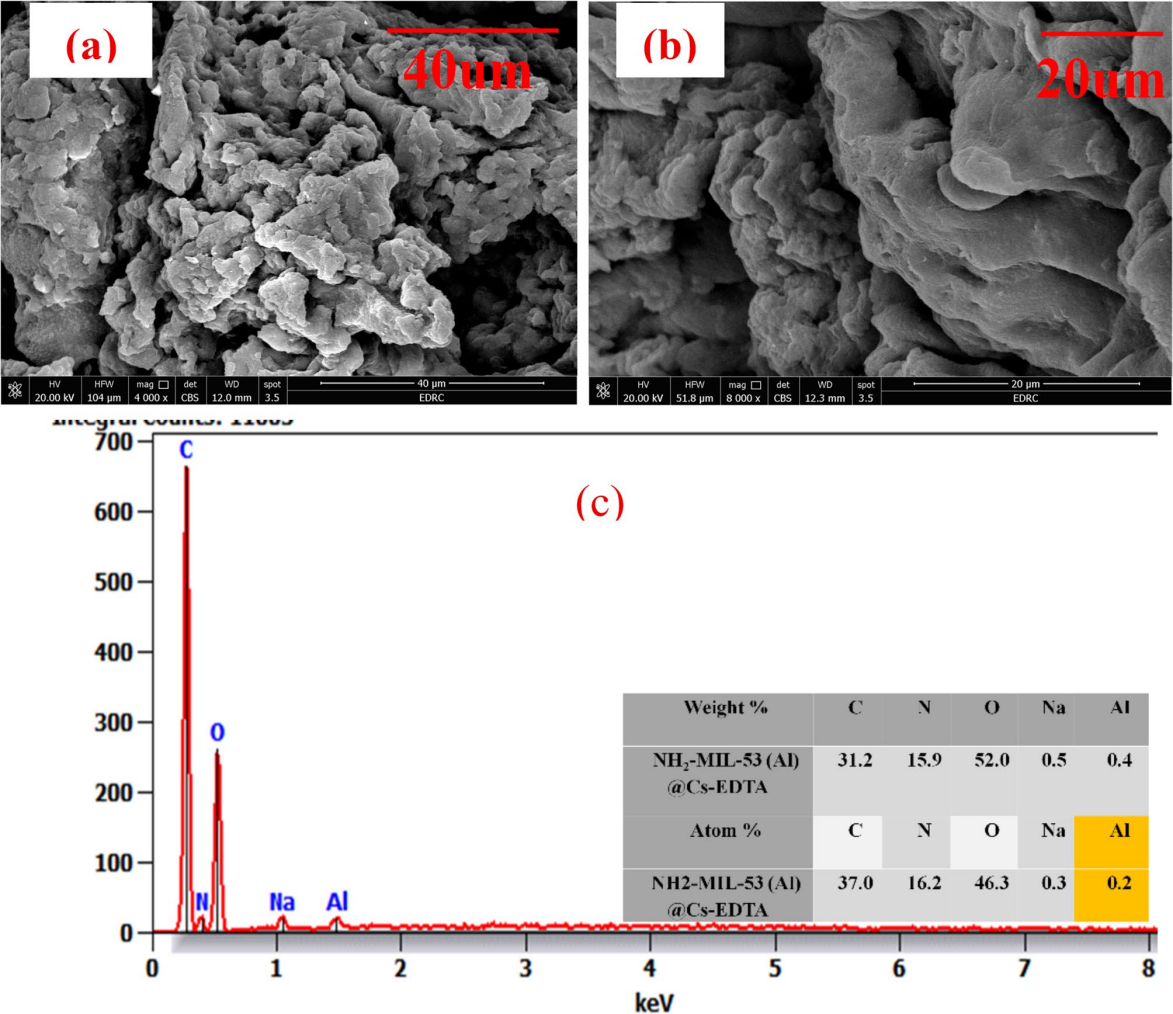
SEM image of NH_2_-MIL-53 (Al) @CS-EDTA with (**a**) low magnification, (**b**) high magnification and (**c**) EDX analysis of NH_2_-MIL-53 (Al) @CS-EDTA.

**Fig. 5:**
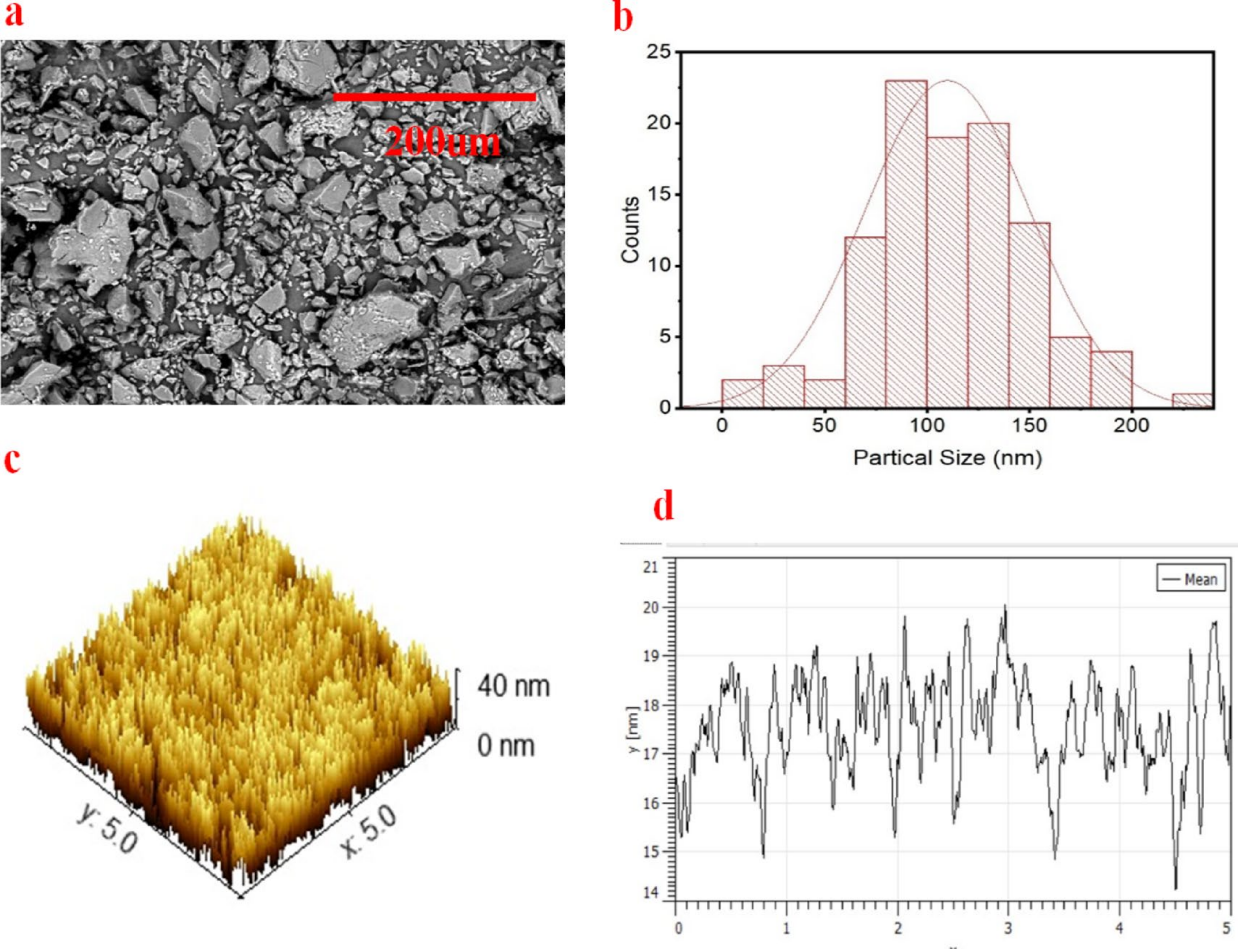
(**a**) The front view of SEM images at different magnification 200×, (**b**) plot of histogram of grain size distribution, (**c**) roughness of prepared material NH_2_-MIL-53 (Al) @ CS-EDTA, and (**d**) height versus distance.

The morphological characteristics of the synthesized materials—CS-EDTA, NH₂-MIL-53(Al), and the composite NH₂-MIL-53(Al)@CS-EDTA—were investigated using scanning electron microscopy (SEM), while their elemental compositions were determined through energy-dispersive X-ray spectroscopy (EDX). The obtained micrographs and spectra are presented in Figs. 2, 3, 4. The SEM micrographs of CS-EDTA exhibit a heterogeneous and irregular surface morphology with agglomerated flake-like particles of various sizes. The surface appears rough and non-uniform, suggesting structural modification of the chitosan matrix after EDTA functionalization. The observed morphology may enhance the accessibility of active sites and facilitate interactions with adsorbate species. In contrast, the NH₂-MIL-53(Al) sample exhibits a heterogeneous morphology composed of densely distributed fine particles with noticeable agglomeration. The particles are irregularly arranged, forming clusters of different sizes and resulting in a rough surface texture. These features are indicative of the characteristic morphology typically associated with MOF materials typical of metal-organic frameworks and confirm the successful formation of the MOF with its characteristic rigid framework. The observed crystal-like features are consistent with the successful formation of the MOF phase. Upon combining the MOF with the CS-EDTA matrix, a significant transformation in surface morphology is observed. The NH₂-MIL-53(Al)@CS-EDTA composite displays a heterogeneous, rough, and porous surface with aggregated structures and irregular grain distribution. The embedding of MOF particles within the polymeric network leads to the development of microcavities and surface protrusions that enhance the surface area. This structural complexity is favorable for adsorption applications, as it increases the availability of active sites and promotes the diffusion of metal ions into the adsorbent structure. Further characterization of the composite’s surface features is provided in Fig. 5, which offers complementary analysis at multiple levels. The SEM image at low magnification (Fig. 5A) reveals a densely packed surface with random grain distribution and extensive aggregation, highlighting the multi-phase nature of the material. These morphological features suggest the coexistence of polymeric and crystalline domains within the composite matrix. The grain size analysis (Fig. 5B) suggests a heterogeneous particle-size distribution within the nanoscale range across both localized and extended regions. The polydispersity in grain size is a consequence of the hybrid synthesis approach and contributes to the heterogeneity of the adsorption environment. The surface roughness distribution (Fig. 5C) further illustrates the textured nature of the composite. The variations in height across the scanned area reveal a complex surface topology, which plays a key role in increasing the effective surface area of the material. Figure 5D, which plots the height versus horizontal distance, suggests the presence of multi-scale surface elevations and depth variations, offering further evidence of the composite’s structural heterogeneity. EDX analysis of the individual and composite materials complements the SEM findings. The CS-EDTA sample shows elemental peaks for carbon, oxygen, nitrogen, and traces of chlorine, consistent with the presence of organic components and EDTA functionalities. NH₂-MIL-53(Al) displays dominant aluminum peaks, in addition to nitrogen and oxygen, suggesting the coordination between Al³⁺ ions and the organic linker. The composite spectrum suggests the simultaneous presence of all expected elements from both components, thereby verifying the successful integration of the MOF into the polymeric framework. No extraneous elements or contamination were detected, suggesting the chemical purity of the synthesized material. Overall, the morphological and compositional analyses demonstrate that NH₂-MIL-53(Al)@CS-EDTA possesses a highly textured, heterogeneous surface with a rough and heterogeneous surface morphology, both of which are critical features for efficient adsorption. These properties, in combination with the chemical stability and elemental uniformity of the composite, underline its potential as a promising adsorbent for water treatment applications.

SEM characterization was primarily employed to provide a qualitative comparison of the morphological characteristics of the synthesized materials. Therefore, the interpretation focuses on observable features such as particle aggregation, surface texture, and morphological heterogeneity, while the structural identification of the composite is supported by the complementary FTIR, XRD, BET, TGA, and EDX analyses.

#### X-ray diffraction analysis (XRD)

**Fig. 6:**
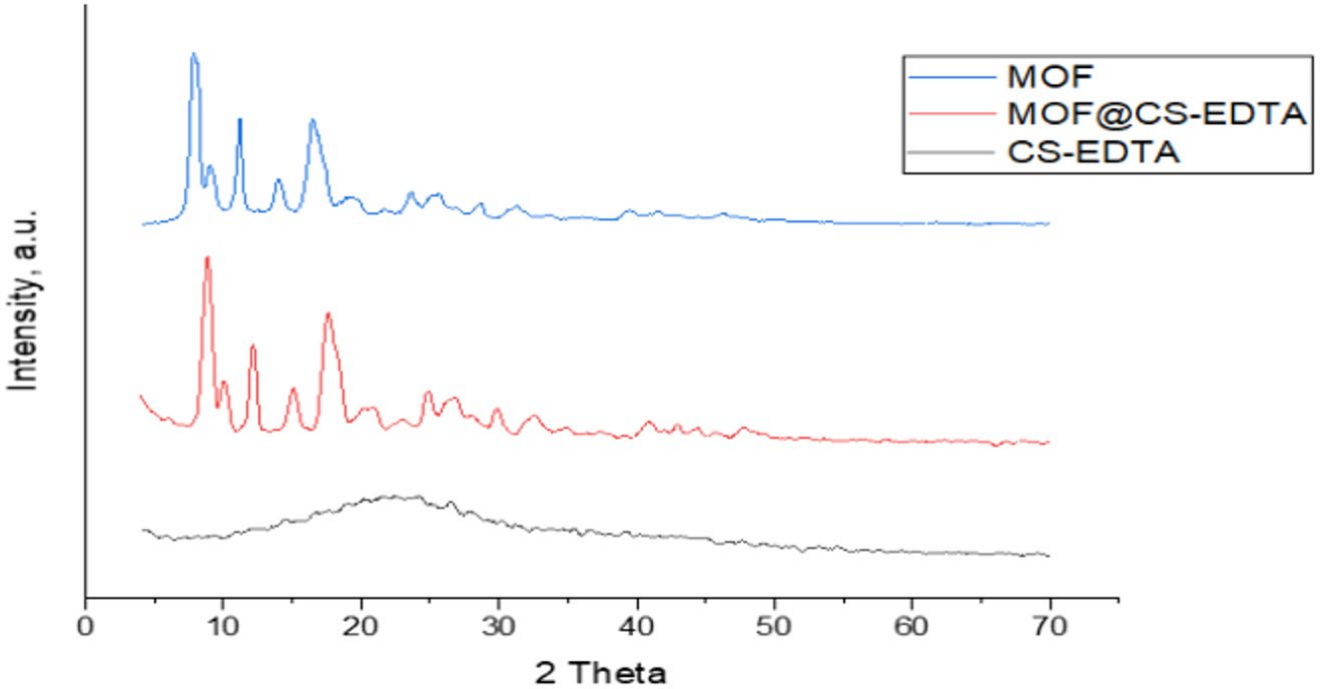
XRD of MOF, MOF@CS-EDTA and CS-EDTA.

The crystalline nature and structural integration of the synthesized materials—NH₂-MIL-53(Al), CS-EDTA, and the composite NH₂-MIL-53(Al)@CS-EDTA—were examined using X-ray diffraction (XRD) analysis, as illustrated in Fig. 6. The XRD pattern of NH₂-MIL-53(Al) displays a series of sharp and well-defined diffraction peaks, indicating a high degree of crystallinity. These peaks are characteristic of the metal-organic framework structure, consistent with previously reported patterns for aluminum-based MOFs, such as those with MIL-53 or MIL-101 architectures. The presence of these distinct peaks suggests the successful synthesis of the MOF with a preserved crystalline framework. In contrast, the XRD pattern of the CS-EDTA sample reveals a broad hump centered around 2θ ≈ 20°, which is a hallmark of amorphous materials. This is typical of polymeric substances like chitosan and EDTA, which lack long-range order and exhibit low crystallinity. The absence of any sharp peaks further suggests the disordered nature of this material. For the composite sample NH₂-MIL-53(Al)@CS-EDTA, the XRD pattern shows a combination of features from both components. The diffraction pattern retains several of the sharp peaks associated with the crystalline MOF, though with slightly reduced intensity and some peak broadening. This suggests that while the MOF structure is preserved within the composite, there may be partial coverage or interaction with the amorphous polymer matrix, leading to a modest decrease in crystallinity. Additionally, the broad amorphous peak at ~ 20°—previously observed in CS-EDTA—also appears in the composite, suggesting the presence of the polymeric matrix. Importantly, the fact that the MOF’s characteristic peaks remain visible indicates that the integration process did not disrupt the internal framework of the MOF. Rather, the composite formation appears to involve a physical embedding or partial surface interaction, rather than complete structural modification. These findings collectively confirm the successful formation of the NH₂-MIL-53(Al)@CS-EDTA hybrid material, in which the crystalline integrity of the MOF is largely retained while being embedded within an amorphous chitosan-EDTA matrix. This structural configuration is advantageous for adsorption applications, as it combines the stability of the MOF with the functional groups and flexibility of the biopolymer matrix.

#### Point zero charge (PZC)

**Fig. 7:**
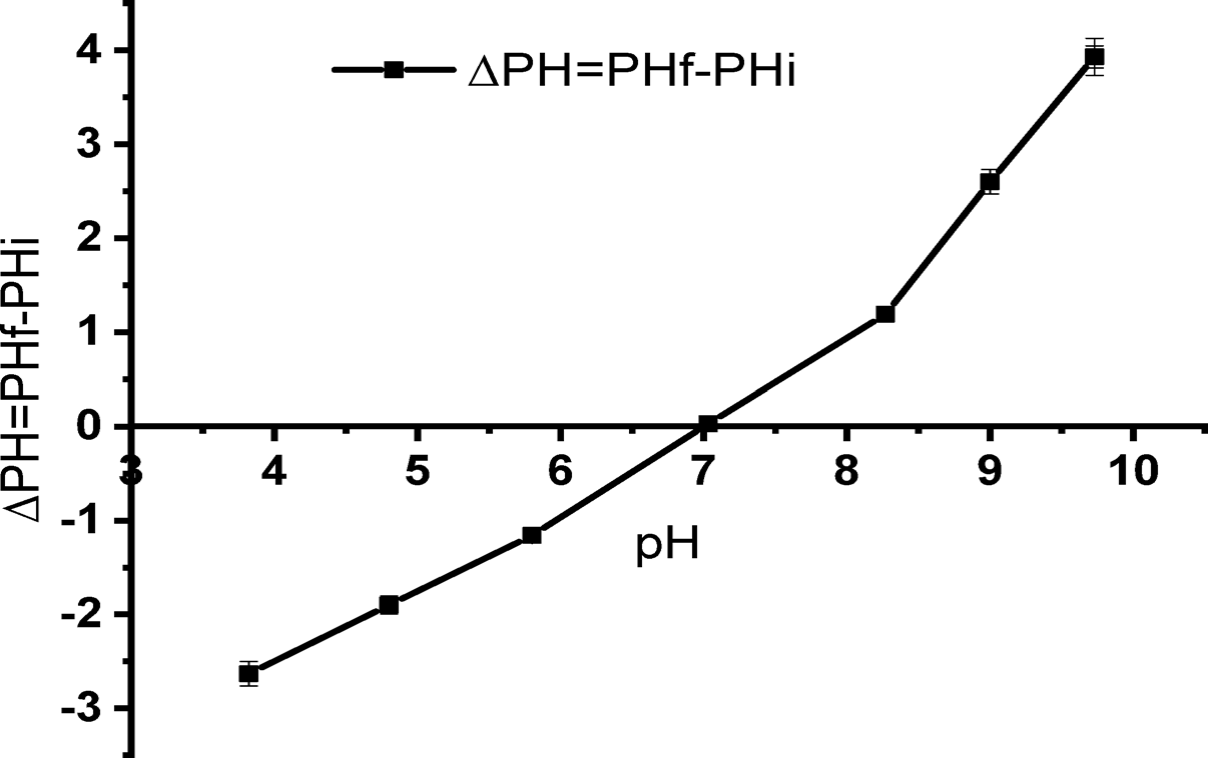
Determination of the point of zero charge (pHPZC) of the NH₂-MIL-53(Al)@CS-EDTA nanocomposite as a function of the initial solution pH. Experimental conditions: 0.01 M NaCl background electrolyte, temperature 298 ± 1 K, and equilibrium time of 24 h.

The point of zero charge (PZC) is a critical parameter that determines the surface charge behavior of adsorbent materials in aqueous environments. For the synthesized NH₂-MIL-53(Al)@CS-EDTA composite, the PZC was experimentally determined to evaluate its surface charge properties under varying pH conditions. The measurement was performed using a salt addition method, in which 10 mL aliquots of 0.01 mol·L^−1^ NaCl solution were placed in multiple beakers, each containing 10 mg of the composite material. The pH values of the solutions were initially adjusted to a range from 2 to 12 using dilute HCl and NaOH solutions. After adding the adsorbent, the mixtures were left under constant agitation for 24 h at room temperature (298 K), ensuring equilibrium between the solid and liquid phases. The initial and final pH values were recorded, and the change in pH (ΔpH) was plotted as a function of the initial pH, as presented in Fig. 7. The resulting curve displayed a crossover point at pH 7.1, which corresponds to the point of zero charge of the NH₂-MIL-53(Al)@CS-EDTA surface. At this specific pH, the net surface charge of the composite is neutral, meaning the number of positive and negative surface sites are balanced. Below the PZC (pH < 7.1), the surface of the composite becomes positively charged, due to the protonation of amine and hydroxyl groups present in the chitosan and the MOF structure. This protonation facilitates electrostatic attraction between the surface and negatively charged species, such as anions. Conversely, at pH values higher than the PZC (pH > 7.1), the surface acquires a negative charge, primarily due to the deprotonation of functional groups such as –COOH and –OH. This shift in charge polarity leads to electrostatic repulsion of anionic species but enhances the adsorption affinity for cationic metal ions, including Cu²⁺, Ni²⁺, and Fe²⁺. Therefore, understanding the PZC is essential in optimizing the adsorption process, particularly in selecting the appropriate pH conditions to maximize removal efficiency. The identification of a PZC at 7.1 aligns well with similar biopolymer–MOF composites reported in literature, suggesting that the hybrid structure maintains a balance of acidic and basic functional groups on the surface. This feature allows the material to be versatile in its interaction with various ionic species, depending on the pH of the solution. But in the presence of EDTA, metal ion adsorption is expected even at low pH values. This is because EDTA contains active functional groups such as carboxyl (–COOH) and amine (–NH₂), which can strongly chelate metal ions (e.g., Cu²⁺, Fe³⁺, Ni²⁺). Therefore, the adsorption process in EDTA-modified materials is mainly driven by chemical complexation rather than electrostatic attraction, making it less dependent on pH compared to unmodified materials (32). Despite the positive surface charge at pH values below the PZC, significant adsorption of Cu²⁺, Fe²⁺, and Ni²⁺ was observed at pH 6. This indicates that electrostatic repulsion is not the dominant factor governing adsorption under these conditions. Instead, chemical complexation between metal ions and the active functional groups (–COOH and –NH₂) of the EDTA–chitosan matrix plays the primary role. These donor atoms coordinate with metal ions through lone-pair electron donation, enabling effective binding even when the overall surface charge is positive.

#### Surface area measurements

**Fig. 8:**
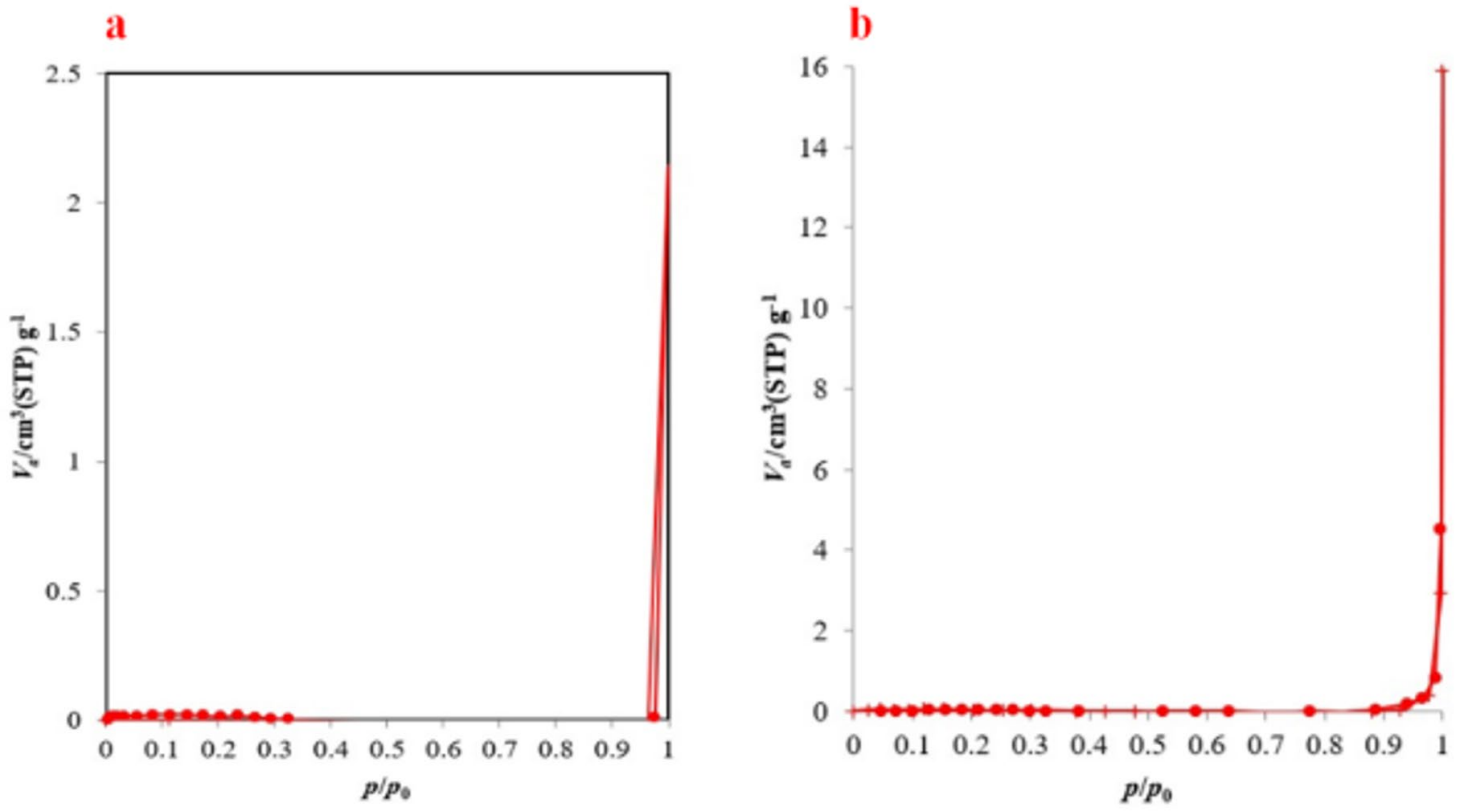
Nitrogen adsorption–desorption isotherms (a) CS-EDTA (b) NH_2_-MIL-53 (Al) @ CS-EDTA.

The surface area and porosity characteristics of the synthesized materials were investigated using nitrogen adsorption–desorption isotherms at 77 K. The isotherm profiles for both CS-EDTA and NH₂-MIL-53(Al)@CS-EDTA are shown in Fig. 8.

The nitrogen adsorption–desorption isotherm of NH₂-MIL-53(Al)@CS-EDTA exhibits a Type IV profile with a distinct hysteresis loop, indicating the occurrence of capillary condensation during nitrogen adsorption–desorption. Such hysteresis behavior is commonly associated with complex pore networks and textural heterogeneity rather than serving as a direct indicator of pore-size classification. According to the BJH analysis, the average pore diameter of the composite is approximately 70 nm, which falls within the macroporous range based on the IUPAC pore-size classification. Therefore, the observed Type IV hysteresis is interpreted as reflecting the hybrid textural characteristics arising from the integration of NH₂-MIL-53(Al) within the CS-EDTA matrix, rather than as direct evidence of its pore-size classification.

A comparative analysis of surface textural parameters, as summarized in Table 1, reveals a marked enhancement in the textural properties upon incorporation of NH₂-MIL-53(Al) into the CS-EDTA matrix. The specific surface area (BET) increased significantly from 0.0078 m²/g for CS-EDTA to 0.18 m²/g for the composite, suggesting the contribution of the incorporated MOF phase to the overall structure. It should be noted that the BET analysis in this study was performed for CS-EDTA and the final NH₂-MIL-53(Al)@CS-EDTA composite. Likewise, the monolayer capacity (Vm) rose from 0.0018 to 0.04 cm³(STP)/g, while the total pore volume increased modestly from 0.0024 to 0.003 cm³/g. Interestingly, the average pore diameter experienced a substantial reduction, from 1228.4 nm in CS-EDTA to 70 nm in the composite. This substantial decrease in the average pore diameter indicates a significant modification of the textural properties of the composite following the incorporation of NH₂-MIL-53(Al). The observed changes are likely associated with structural rearrangement and the interaction between the polymer matrix and the embedded MOF particles, resulting in a more compact and heterogeneous pore structure compared with pristine CS-EDTA. The larger pores observed in pristine CS-EDTA are likely related to the irregular packing of the polymer chains, whereas incorporation of the MOF modifies the overall textural characteristics of the composite. Although incorporation of NH₂-MIL-53(Al) increased the BET surface area compared with CS-EDTA, the absolute BET surface area of the composite remained relatively low (0.18 m² g^−1^). Therefore, the adsorption performance cannot be attributed solely to a high specific surface area or extensive physical porosity. Instead, the adsorption behavior is more reasonably associated with the high density of accessible chelating functional groups (–NH₂, –COOH, and –OH) introduced by the chitosan–EDTA matrix together with the embedded NH₂-MIL-53(Al) framework, which collectively promote strong coordination interactions with metal ions. In addition, previous studies have shown that the measured BET surface area of MOF–polymer composites may be influenced by factors such as partial pore blocking by the polymer matrix, relatively low MOF loading, and sample activation (degassing) conditions prior to nitrogen adsorption measurements. Accordingly, the adsorption performance of the present composite is discussed primarily in terms of surface chemistry and functional-group-mediated complexation rather than high BET surface area alone.

It should be noted that the average pore diameter reported in this study was obtained using the BJH method and should be regarded as an average textural parameter for the hybrid composite. In polymer–MOF composites, this value may also reflect interparticle voids and structural heterogeneity rather than intrinsic MOF pores alone. Accordingly, the pore-size discussion has been revised to conform with the IUPAC pore classification.

**Table 1:** BET, pore volume, and average pore diameter of CS-EDTA and NH_2_-MIL-53 (Al) @CS-EDTA nanocomposite.

Parameter	Unit	Chitosan-EDTA	NH_2_-MIL-53 (Al) @CS-EDTA
V_m_	cm^3^ g^− 1^	0.0018	0.04
BET	m^2^ g^− 1^	0.0078	0.18
Total pore volume	cm^3^ g^− 1^	0.0024	0.003
Average pore diameter	nm	1228.4	70

#### Thermogravimetric analysis (TGA)

**Fig. 9:**
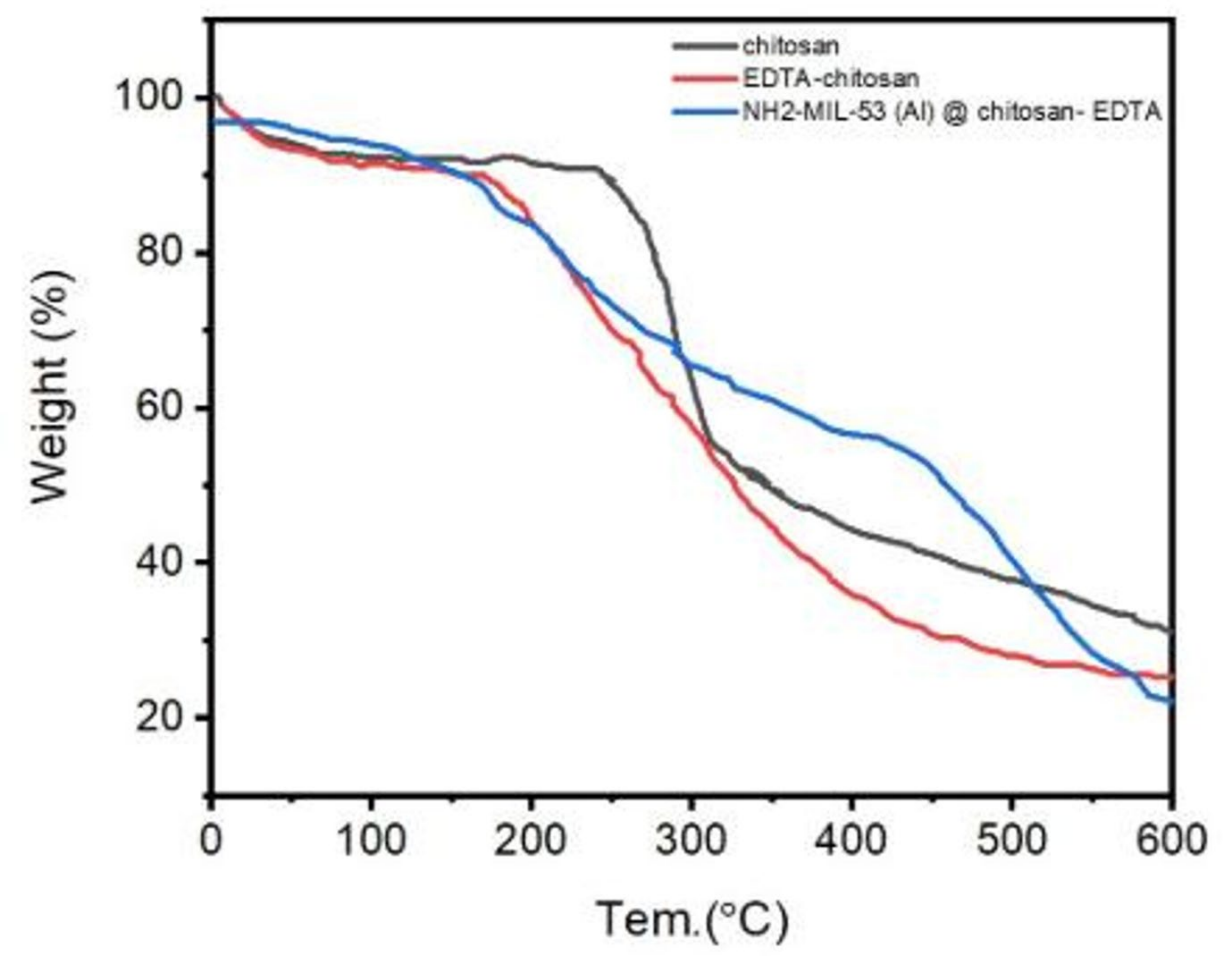
Thermogravimetric analysis (TGA) curves of chitosan, CS-EDTA, and NH₂-MIL-53(Al)@CS-EDTA recorded under a nitrogen atmosphere over the temperature range of 25–800 °C at a heating rate of 10 °C min^−1^.

Thermogravimetric analysis (TGA) was conducted to evaluate the thermal stability and decomposition behavior of the individual and composite materials, including pristine chitosan, CS-EDTA, and NH₂-MIL-53(Al)@CS-EDTA. The TGA thermograms for these samples are shown in Fig. 9. Pristine chitosan exhibits a two-step thermal degradation pattern. The first weight loss, observed between 100 °C and 150 °C, is attributed to the evaporation of physically adsorbed moisture and residual solvents. This is followed by a major degradation stage occurring between 200 °C and 350 °C, corresponding to the depolymerization and decomposition of the chitosan backbone. The final degradation phase, which occurs above 350 °C, involves carbonization and the formation of stable char residues. The total residual mass at the end of the heating process is approximately 30%, indicating the semi-crystalline nature of chitosan and its moderate thermal resilience. In comparison, the CS-EDTA sample is consistent with lower thermal stability. Initial degradation begins slightly earlier, around 180 °C, and extends to approximately 300 °C. The reduction in thermal stability is attributed to the incorporation of EDTA, which disrupts the intramolecular hydrogen bonding network of chitosan and introduces thermally labile carboxylate and amine groups. The overall residue after decomposition is reduced to about 20–25%, suggesting the destabilizing influence of EDTA on the polymeric matrix. Conversely, the thermal behavior of the NH₂-MIL-53(Al)@CS-EDTA composite indicates improved thermal resistance compared with the chitosan-based materials, suggesting successful incorporation of the MOF phase within the polymeric matrix. The onset of significant weight loss is shifted to higher temperatures, and the degradation proceeds more gradually. This behavior reflects the thermal robustness of the MOF component, which likely acts as a heat-resistant scaffold embedded within the polymeric matrix. Moreover, the presence of strong interactions between the metal-organic framework and functional groups of chitosan and EDTA—such as hydrogen bonding and possible coordination with Al³⁺ centers—may further stabilize the composite structure against thermal decomposition. The composite also retains a relatively high residual mass of approximately 25–30%, which is comparable to or slightly higher than the individual components. This supports the notion that the inclusion of NH₂-MIL-53(Al) reinforces the thermal resistance of the material, acting as a barrier to thermal transport and degradation. In summary, the TGA results suggest that the thermal stability of the chitosan-based matrix is significantly improved upon the incorporation of the NH₂-MIL-53(Al) framework. This enhancement is crucial for practical applications, especially in adsorption processes involving elevated temperatures or thermal regeneration cycles.

It should be noted that the thermogravimetric analysis in the present study was performed to evaluate the thermal behavior of chitosan, CS-EDTA, and the final NH₂-MIL-53(Al)@CS-EDTA composite. Although thermogravimetric characterization of pristine NH₂-MIL-53(Al) would provide an additional reference for evaluating thermal changes associated with composite formation and polymer loading, such analysis was beyond the scope of the present work.

### Adsorption performance of Cu²⁺, Ni²⁺, and Fe²⁺ ions

#### Effect of adsorbent dosage

**Fig. 10:**
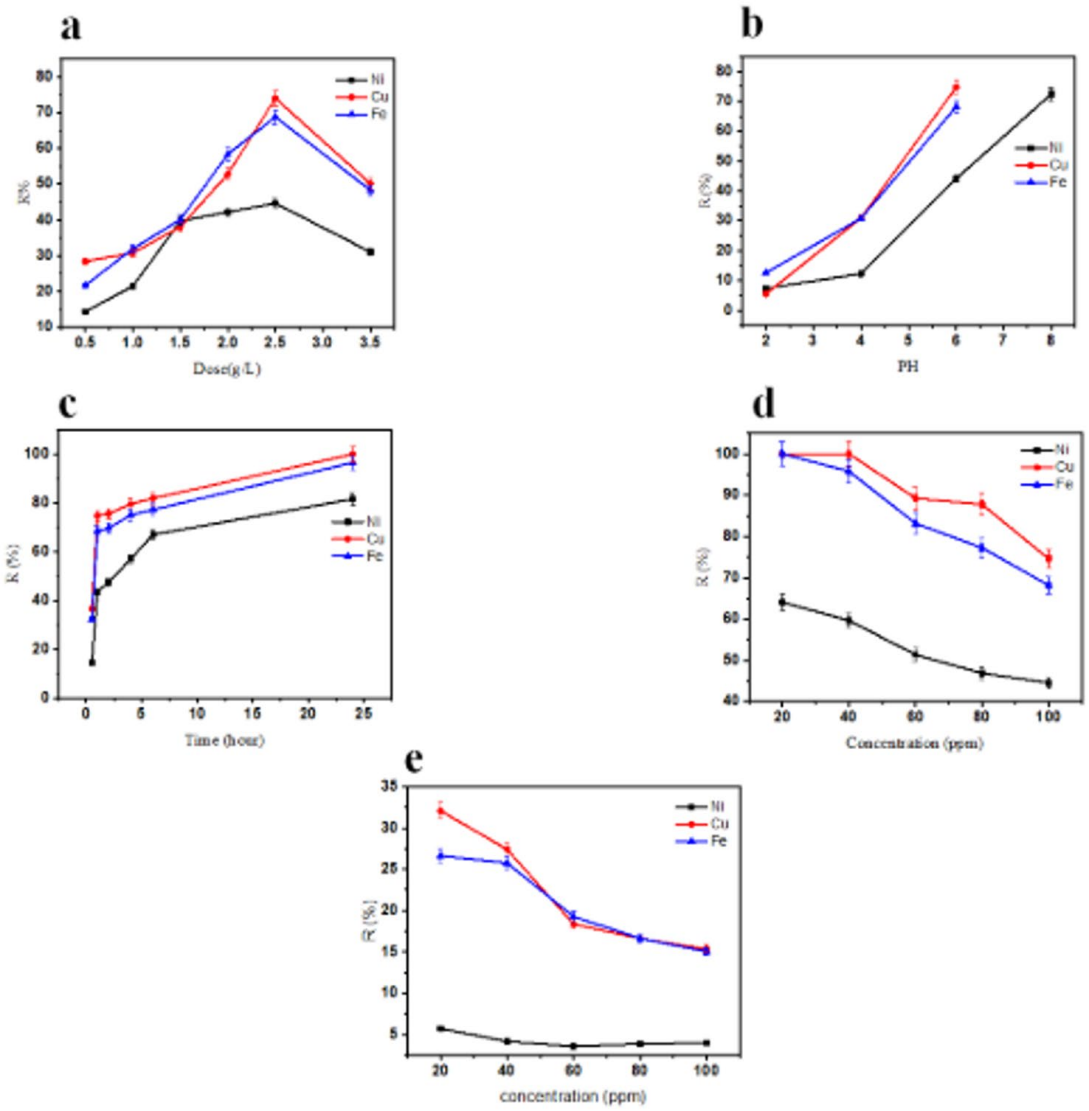
Effect of different adsorption parameters onto the Cu²⁺, Ni²⁺, and Fe²⁺ ions removal efficiency using NH_2_-MIL-53 (Al) @CS-EDTA adsorbent; (**a**) adsorbent dose, (**b**) pH of the aqueous solution, (**c**) contact time, (**d**) ions concentration and (**e**) competitive ions.

The dosage of adsorbent is a key parameter that directly influences the adsorption efficiency, as it dictates the number of available active sites on the material’s surface. In this study, the adsorbent dosage of NH₂-MIL-53(Al)@CS-EDTA was varied from 0.5 to 3.5 g L^−1^ to evaluate its impact on the removal of Cu²⁺, Ni²⁺, and Fe²⁺ ions under constant conditions of pH, contact time, and initial metal ion concentration. As illustrated in Fig. 10a, the adsorption efficiency increased progressively with adsorbent dosage up to an optimum value of 2.5 g L^−1^, where maximum removal efficiencies of 68% for Cu²⁺, 46% for Ni²⁺, and 68% for Fe²⁺ were achieved. This enhancement is attributed to the increased number of available active sites for ion binding. However, further increase in dosage beyond 2.5 g L^−1^ resulted in a slight decline or plateau in adsorption efficiency. This may be due to particle agglomeration at higher dosages, leading to a reduction in effective surface area and mass transfer limitations. Thus, 2.5 g L^−1^ was selected as the operational adsorbent dosage for subsequent adsorption studies. It should be noted that the dosage screening experiment was performed using a fixed contact time of 1 h to compare the relative effect of adsorbent amount under identical conditions. Although the kinetic study showed that equilibrium was reached after 24 h, a substantial fraction of metal uptake occurred during the initial adsorption stage, supporting the use of this dosage for comparative adsorption investigations.

#### Influence of initial pH

The pH of the aqueous solution is known to play a decisive role in metal ion adsorption by influencing both the surface charge of the adsorbent and the speciation of metal ions in solution. In this study, the pH was varied from 2 to12 to evaluate its effect on the adsorption of Cu²⁺, Ni²⁺, and Fe²⁺ ions. Figure 10b shows that the adsorption efficiency increased with rising pH for all three ions, reaching a maximum at pH 6 for Cu²⁺ and Fe²⁺. At lower pH levels, protonation of surface functional groups competes with metal ions for active sites, reducing adsorption due to electrostatic repulsion. As pH increases, deprotonation of amino and hydroxyl groups on the adsorbent surface enhances electrostatic attraction toward the positively charged metal ions. However, it is critical to note that increasing pH beyond 6 may lead to the formation of metal hydroxide precipitates, especially for Cu²⁺ and Fe²⁺, which interferes with the adsorption process and falsely inflates removal efficiency. Therefore, any apparent increase in metal removal efficiency above pH 6.5 may partially result from precipitation rather than true surface adsorption. To ensure reliable interpretation of adsorption behavior, all subsequent experiments were conducted at pH 6, where hydroxide formation is minimal, and adsorption reflects primarily chemical binding processes. Therefore, pH 6 was selected as the upper operational limit to avoid precipitation and ensure reliable adsorption data. For Ni²⁺, the increase in adsorption was also observed with pH, but the trend was less pronounced, possibly due to its different complexation behavior in aqueous media. It should also be noted that Fe²⁺ ions may undergo oxidation to Fe³⁺ followed by hydroxide precipitation under alkaline conditions. Consequently, the apparent removal observed at elevated pH values may not be exclusively attributed to adsorption. Therefore, the removal observed at high pH values should be interpreted with caution, as both adsorption and precipitation mechanisms may contribute to metal removal. Accordingly, pH 6 was selected as the operating pH for all subsequent adsorption experiments to minimize precipitation effects and ensure that the observed metal removal predominantly reflects adsorptive interactions between the metal ions and the NH₂-MIL-53(Al)@CS-EDTA composite. It should also be noted that the residual iron concentration was determined as total dissolved iron by flame atomic absorption spectroscopy (AAS), which does not distinguish between Fe²⁺ and Fe³⁺ species. Therefore, the oxidation state of iron was not monitored during the adsorption experiments. Although freshly prepared Fe²⁺ stock solutions were used and the adsorption experiments were completed within the experimental timeframe, partial oxidation of Fe²⁺ under aerobic conditions cannot be completely excluded. Consequently, the reported results should be interpreted as representing the overall removal of dissolved iron under the investigated experimental conditions rather than the exclusive removal of Fe²⁺.

#### Influence of contact time

The effect of contact time on the adsorption efficiency was investigated by exposing a fixed dosage (0.05 g, equivalent to 2.5 g L^–1^) of the NH₂-MIL-53(Al)@CS-EDTA adsorbent to solutions of Cu²⁺, Ni²⁺, and Fe²⁺ over time intervals ranging from 0.5 to 24 h. Figure 10c displays the adsorption profiles. The results show a rapid initial uptake within the first hour, indicative of abundant available active sites and a strong driving force for mass transfer. Over time, the adsorption rate gradually slowed as the active sites became saturated. After 24 h, the removal efficiencies reached approximately 100% for Cu²⁺, 96% for Fe²⁺, and 81% for Ni²⁺, reflecting excellent adsorption kinetics for Cu²⁺ and Fe²⁺ and a relatively lower affinity for Ni²⁺. In contrast, the comparatively lower uptake of Ni²⁺ may be due to weaker binding interactions or slower diffusion into the internal pores.

####  Influence of initial metal ion concentration

To assess the adsorbent’s performance at different pollution levels, the initial concentrations of Cu²⁺, Ni²⁺, and Fe²⁺ were varied from 20 to 100 mg L^–1^. Figure 10d illustrates the relationship between ion concentration and adsorption efficiency. For Cu²⁺ and Fe²⁺, the adsorption capacity increased slightly with rising concentration, consistent with the increase in the concentration gradient which enhances mass transfer to the surface. However, at higher concentrations, the limited number of active sites on the adsorbent begins to restrict further uptake, resulting in a plateau. Interestingly, Ni²⁺ adsorption showed minimal variation across the tested concentration range. This indicates either a low binding affinity between Ni²⁺ ions and the active sites of NH₂-MIL-53(Al)@CS-EDTA, or fast saturation kinetics at even low concentrations. This finding highlights the selectivity of the composite material, which exhibits higher affinity for Cu²⁺ and Fe²⁺ than for Ni²⁺ under the same experimental conditions.

#### Influence of competitive ions

To assess the practical applicability of the synthesized NH₂-MIL-53(Al)@CS-EDTA composite in real wastewater environments, the effect of competitive ions was investigated. These ions, which commonly coexist in industrial effluents, can interfere with adsorption processes by occupying active binding sites. In this study, the initial concentrations of competing ions were varied from 20 to 100 mg L^–1^, and the results are presented in Fig. 10e. Interestingly, the adsorption capacity of the composite for Cu²⁺ and Fe²⁺ ions increased slightly in the presence of the investigated competing ions, while the adsorption of Ni²⁺ remained nearly constant. Although this observation differs from the competitive inhibition commonly expected in multicomponent adsorption systems, the present data do not provide direct evidence to identify the origin of this behavior. Therefore, the observed enhancement should be regarded as an empirical observation under the investigated experimental conditions rather than confirmation of a synergistic adsorption mechanism. The observed behavior is most reasonably interpreted within the framework of the solution chemistry and the multifunctional nature of the developed composite under the investigated experimental conditions, while the specific contribution of the individual interactions cannot be distinguished from the present data. It should also be noted that the present competitive adsorption study was limited to coexisting heavy metal ions, namely Cu²⁺, Fe²⁺, and Ni²⁺, in binary and ternary systems. Other matrix components commonly present in real wastewater, such as organic dyes, antibiotics, dissolved salts, and variations in ionic strength, may also influence adsorption performance through competitive binding, pore blockage, electrostatic screening, or changes in metal speciation. Therefore, further studies using more complex wastewater matrices are recommended to better assess the practical applicability of NH₂-MIL-53(Al)@CS-EDTA under real operating conditions.

#### Maximum adsorption

**Fig. 11:**
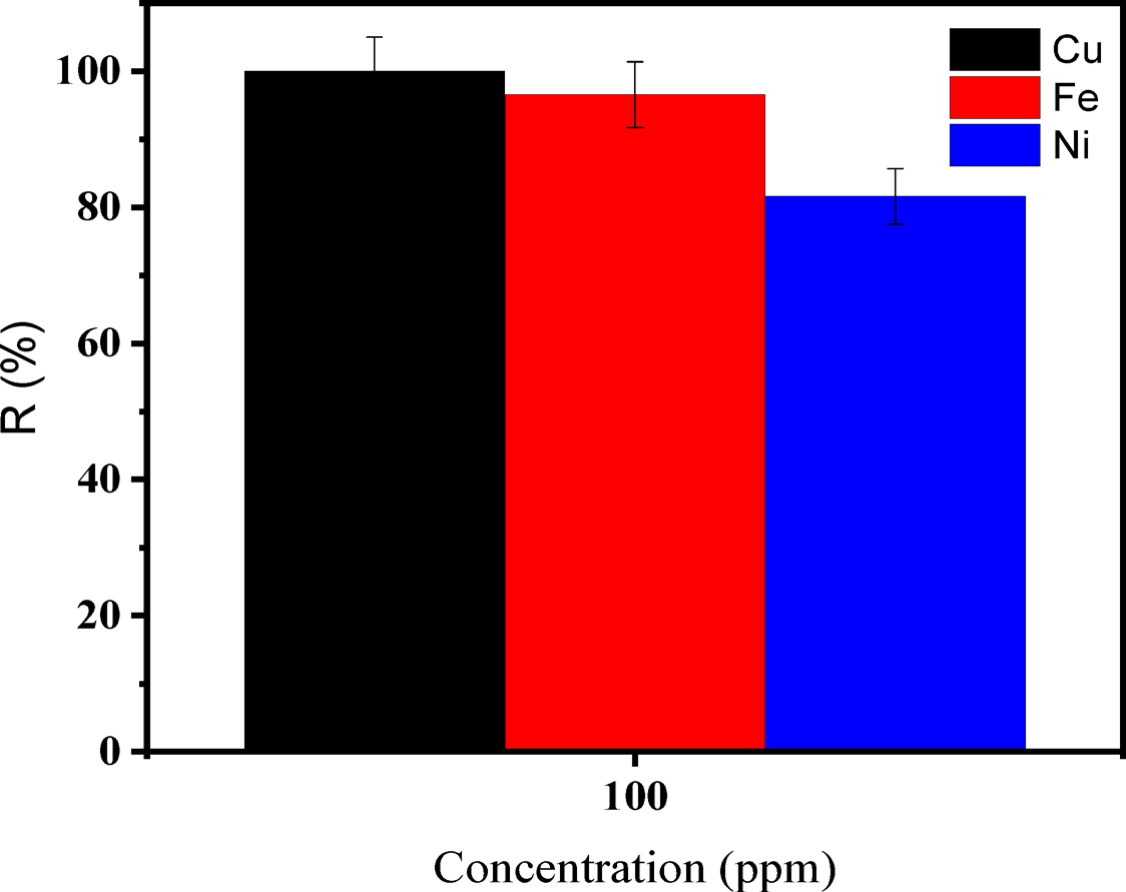
Maximum adsorption of NH_2_-MIL-53 (Al) @CS-EDTA composite.

After 24 h of contact at an initial metal concentration of 100 ppm, the composite exhibited the highest removal efficiency toward Cu²⁺ (100%), followed by Fe²⁺ (96%), and Ni²⁺ (81%). This trend (Cu²⁺ > Fe²⁺ > Ni²⁺) can be attributed to the higher complexation affinity and stability constants of Cu²⁺ and Fe²⁺ with the functional groups of chitosan and EDTA (–NH₂, –COOH, –OH), as well as the available coordination sites within the NH_2_-MIL-53 (Al) structure. In contrast, Ni²⁺ forms less stable coordination complexes under similar conditions, resulting in comparatively lower adsorption efficiency. The results are illustrated in Fig. 11.

#### Influence of adsorbent chemical composition

**Fig. 12:**
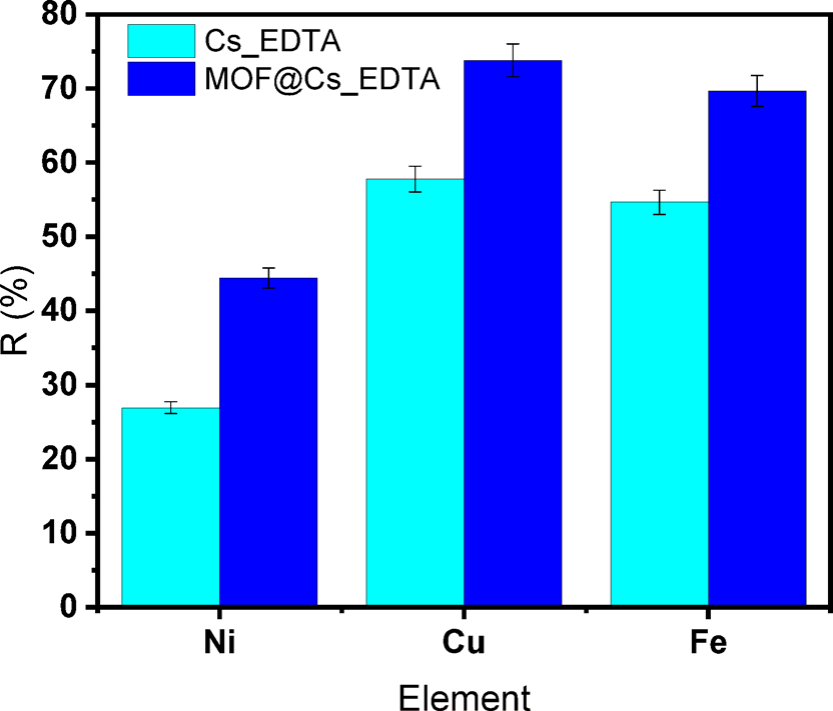
Effect of adsorbent type on the removal efficiency of Cu²⁺, Ni²⁺, and Fe²⁺ ions under the optimized adsorption conditions. Experimental conditions: adsorbent dosage = 2.5 g L^−1^, initial metal ion concentration = 100 mg L^−1^, solution pH = 6.0, contact time = 60 min, temperature = 298 ± 1 K, and agitation speed = 150 rpm. Error bars represent the standard deviation of three independent experiments.

To evaluate the contribution of material composition to adsorption performance, a comparative analysis was conducted between CS-EDTA and the NH₂-MIL-53(Al)@CS-EDTA nanocomposite. Adsorption experiments were carried out under optimized conditions: adsorbent dosage of 2.5 g L^–1^, metal ion concentration of 100 mg L^–1^, contact time of 1 h, and solution pH of 6. The results are illustrated in Fig. 12. The removal efficiencies were significantly higher for the NH₂-MIL-53(Al)@CS-EDTA composite across all three metal ions. This enhanced performance is attributed to the synergistic role of the NH₂-MIL-53(Al) framework and EDTA functional groups, which jointly offer increased adsorption sites, chelation abilities, and structural stability. While EDTA introduces abundant chelating functional groups capable of strongly binding metal ions, the incorporated NH₂-MIL-53(Al) provides additional accessible adsorption sites and structural reinforcement. Therefore, the improved adsorption performance arises mainly from the synergistic combination of chemical functionality and composite architecture rather than from a high BET surface area alone. These results underscore the critical role of functional group integration and structural engineering in improving adsorbent performance, supporting the rational design of the NH₂-MIL-53(Al)@CS-EDTA composite for environmental remediation applications.

### The kinetic studies

The kinetic studies were conducted to elucidate the underlying adsorption mechanism and to evaluate the potential of this material in practical applications. Two kinetic models were employed: the pseudo-first-order and the pseudo-second-order models. The pseudo-first-order kinetic model, which assumes that the rate of adsorption is directly proportional to the number of unoccupied active sites, is described by the following linear equation:

4 $$\:\mathrm{ln}({q}_{e}-{q}_{t})=\mathrm{ln}\left({q}_{e}\right)-{k}_{1}t$$

Where $\:{q}_{e}$ and q_t_ (mg. g^− 1^) are the adsorption capacities at equilibrium and at time t, respectively, of Cu^2+^, Fe^2+^ and Ni^2+^, and k_*1*_ (h^− 1^) is the rate constant of pseudo first-order adsorption model.

The pseudo-second-order kinetic model is widely used to describe adsorption systems involving surface interactions between the adsorbent and the adsorbate. Nevertheless, recent studies have emphasized that agreement with this model alone should not be considered conclusive evidence of chemisorption without complementary physicochemical characterization^33–39^. It is expressed in the linearized form:

5 $$\:\frac{t}{{q}_{t}}=\frac{1}{{k}_{2}{q}_{e}^{2}}+\frac{t}{{q}_{e}}$$

Where *k*_*2*_ (g.mg^−1^h^− 1^) is the rate constant for the pseudo-second-order model.

**Fig. 13:**
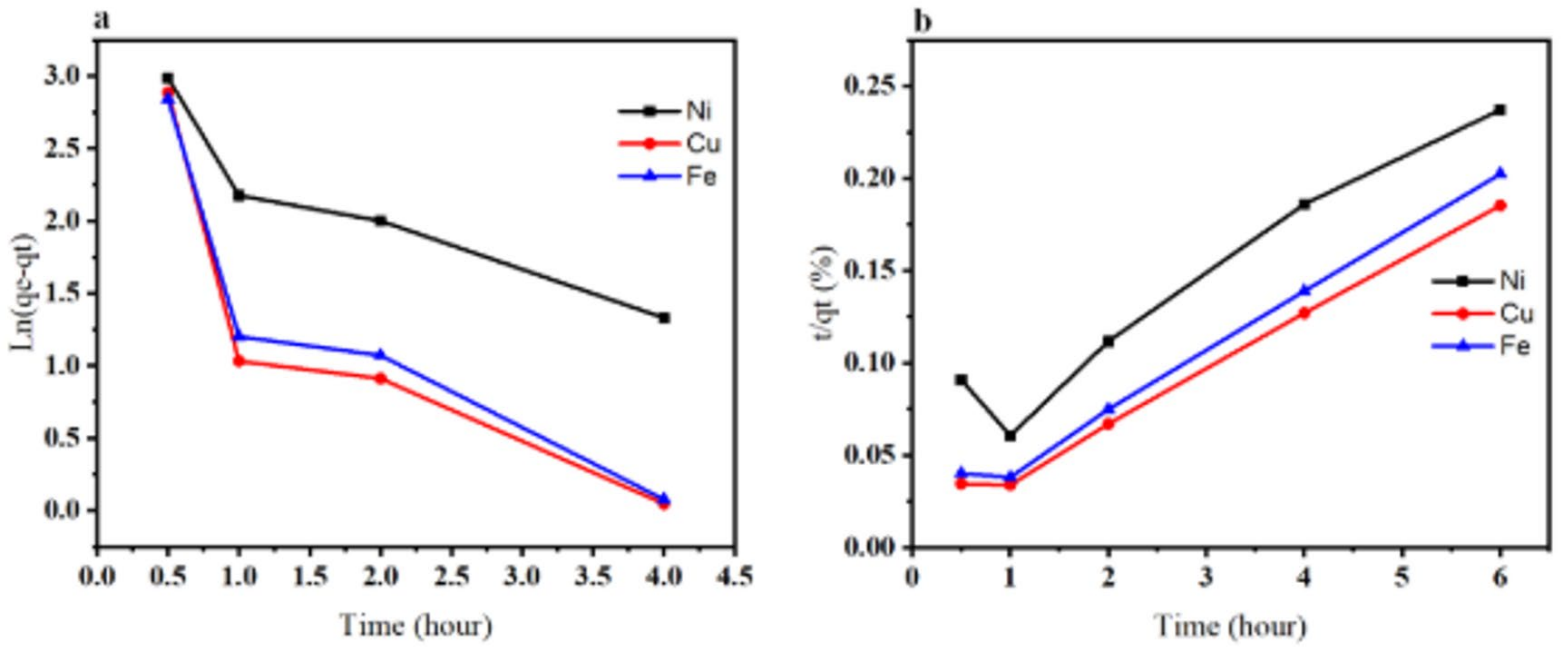
Linear kinetic modeling of the adsorption behavior of Cu²⁺, Fe²⁺, and Ni²⁺ ions onto NH₂-MIL-53(Al)@CS-EDTA composite: (**a**) pseudo-first-order kinetics and (**b**) pseudo-second-order kinetics. Adsorption experiments were carried out under optimized conditions: adsorbent dosage of 0.05 g, metal ion concentration of 100 mg L^−1^, volume 0.02 L, contact time (0.5,1,2,4 and 6) hour, and solution pH of 6. The plots indicate the kinetic model that best describes the experimental adsorption data for each metal ion under the investigated conditions.

Linear fitting was performed for Cu²⁺, Fe²⁺, and Ni²⁺ adsorption onto NH₂-MIL-53(Al)@CS-EDTA. The corresponding kinetic parameters—such as the calculated and experimental adsorption capacities (q_e_ and q_exp_) and correlation coefficients (R^2^)—are summarized in Table 2. The adsorption of Cu²⁺, Fe²⁺, and Ni²⁺ ions onto NH₂–MIL-53(Al)@CS–EDTA was better described by the pseudo-second-order kinetic model than by the pseudo-first-order model, as evidenced by the higher correlation coefficients (R² ≈ 0.94–0.99). Moreover, the equilibrium adsorption capacities calculated from the pseudo-second-order model showed good agreement with the corresponding experimental values. This finding indicates that the adsorption rate is well described by the assumptions of the pseudo-second-order kinetic model under the investigated conditions. However, agreement with this kinetic model alone should not be interpreted as direct evidence that chemisorption is the sole or dominant adsorption mechanism. Instead, the adsorption behavior is more appropriately interpreted in conjunction with the available physicochemical characterization and the proposed adsorption pathways discussed in “Proposed adsorption mechanism of heavy metals onto NH₂-MIL-53(Al)@CS-EDTA”. This interpretation is consistent with recent studies demonstrating that kinetic model fitting alone is insufficient to establish the adsorption mechanism. Therefore, in the present work, the kinetic analysis is interpreted only as being consistent with the proposed adsorption mechanism when considered together with the physicochemical characterization, equilibrium modeling, and the well-established coordination chemistry of the functional groups present in the NH₂–MIL-53(Al)@CS–EDTA composite, rather than as direct proof of chemisorption^33–39^ (Fig. 13).

**Table 2:** Kinetic model parameters for the adsorption of Cu²⁺, Fe²⁺, and Ni²⁺ onto NH₂-MIL-53(Al)@CS-EDTA, based on pseudo-first-order and pseudo-second-order models.

Metal ion	q _exp_ (mg mg^− 1^)	Pseudo-first-order			Pseudo-second-order		
		q_e_ (mg mg^− 1^)	K_1_ (h^−1^)	*R* ^2^	q_e_ (mg mg^− 1^)	K_2_ (g mg^− 1^ h^− 1^)	*R* ^2^
Cu^2+^	32.38	11.4089 ± 0.496	0.6481 ± 0.215	0.7049	34.843 ± 0.0012	0.06779 ± 0.004	0.9928
Fe^2+^	29.16	12.3604 ± 0.498	0.7484 ± 0.216	0.7684	32.247 ± 0.0011	0.06264 ± 0.0039	0.9919
Ni^2+^	25.30	17.948 ± 0.06	0.4077 ± 0.026	0.8602	32.341 ± 0.001	0.01777 ± 0.0036	0.9457

### Adsorption isotherm study

The adsorption equilibrium data for the removal of Cu²⁺, Fe²⁺, and Ni²⁺ ions using the NH₂-MIL-53(Al)@CS-EDTA nanocomposite were analyzed using both Langmuir and Freundlich isotherm models, providing critical insight into the nature and mechanism of the adsorption process. These models help clarify whether the adsorption takes place on a homogeneous or heterogeneous surface and whether it involves monolayer or multilayer interactions. Similar kinetic and equilibrium modeling approaches have been widely used to interpret adsorption mechanisms and evaluate the practical performance of composite adsorbents^40^.

The Langmuir isotherm model assumes that the adsorption process occurs on a homogenous surface with a finite number of identical active sites, forming a monolayer of adsorbed molecules. This model is mathematically described by the equation:

6 $$\:\frac{{C}_{e}}{{q}_{e}}=\frac{{C}_{e}}{{q}_{max}}+\frac{1}{{K}_{Le}{q}_{max}}$$

where C_e_ (mgL^− 1^) and $\:{q}_{e}$ (mg g^− 1^) are the concentrations of the studied heavy metals and the capacity of adsorption, respectively.

The linear fitting of the Langmuir isotherm yielded correlation coefficients (R²) of 0.9895, 0.9831, and 0.9852 for Cu²⁺, Fe²⁺, and Ni²⁺, respectively (Table 3), indicating that the Langmuir equation provides a better mathematical description of the equilibrium adsorption data than the Freundlich model under the investigated experimental conditions. However, it should be emphasized that agreement with the Langmuir model alone is insufficient to conclusively establish monolayer adsorption or the presence of perfectly homogeneous adsorption sites, particularly since the Freundlich model also exhibited reasonably good correlation for some of the investigated metal ions. Recent studies have similarly highlighted that equilibrium isotherm fitting should be interpreted cautiously and should not be used as the sole basis for mechanistic conclusions. Accordingly, the Langmuir model is interpreted here as providing the most appropriate equilibrium description of the experimental data within the studied concentration range rather than direct evidence of a specific adsorption mechanism or surface homogeneity^41–44^.

The Langmuir-derived maximum adsorption capacities (qmax) were 31.8, 28.1, and 21.0 mg g^−1^ for Cu²⁺, Fe²⁺, and Ni²⁺, respectively. The comparatively higher adsorption capacities obtained for Cu²⁺ and Fe²⁺ are consistent with their stronger coordination affinity toward the oxygen- and nitrogen-containing functional groups (–NH₂, –OH, and –COOH) present in the NH₂-MIL-53(Al)@CS-EDTA composite. Nevertheless, this interpretation is based on the known coordination chemistry of these metal ions together with the observed adsorption behavior and should therefore be regarded as a plausible explanation rather than direct experimental confirmation. In agreement with the revised mechanistic discussion presented in “Proposed adsorption mechanism of heavy metals onto NH₂-MIL-53(Al)@CS-EDTA”, definitive identification of the adsorption mechanism would require complementary post-adsorption characterization techniques such as FT-IR, XPS, or elemental mapping^41–44^.

On the other hand, the Freundlich isotherm, an empirical model describing adsorption on heterogeneous surfaces with varying affinities, is expressed as:

7 $$\:\mathrm{ln}{q}_{e}=\mathrm{ln}{k}_{F}+\left(1/n\right)\mathrm{ln}{C}_{e}$$

Where $\:{K}_{F}$ (L/g) represents the Freundlich constant, and $\:1/n\:$ is related to the adsorption strength.

**Fig. 14:**
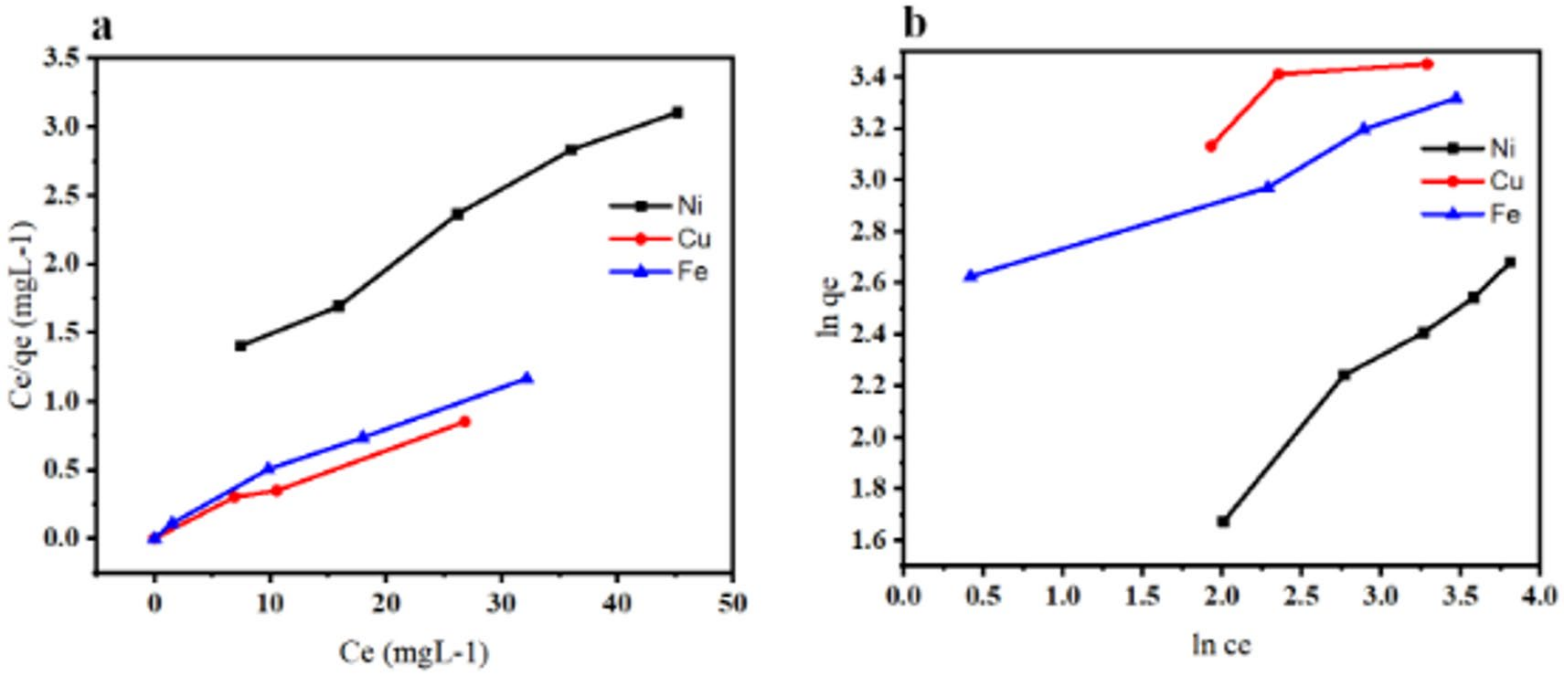
Adsorption isotherms for Cu²⁺, Fe²⁺, and Ni²⁺ ions using NH₂-MIL-53(Al)@CS-EDTA adsorbent: (**a**) Langmuir isotherm model (Ce/qe vs. Ce), (**b**) Freundlich isotherm model (ln qe vs. ln Ce). Adsorption experiments were carried out under optimized conditions: adsorbent dosage of 0.05 g, metal ion concentration of (20,40,60,80and100) mg L^− 1^, volume 0.02 L, contact time of 1 h, and solution pH of 6.

The linear plots of ln q_e_ versus ln Ce, shown in Fig. 14b, yielded lower R² values (0.6606–0.9738), indicating a weaker fit compared to the Langmuir model. However, the values of the heterogeneity factor 1/n for all ions were less than 1, which suggests that the adsorption process remains favorable across the studied concentration range. Although the Freundlich model captured the surface heterogeneity to some extent, its lower correlation coefficients indicate that the Langmuir model provides a better mathematical description of the equilibrium data under the investigated experimental conditions. However, agreement with the Langmuir model alone should not be interpreted as direct evidence of monolayer adsorption or a specific adsorption mechanism. Instead, the adsorption behavior is more appropriately interpreted by considering the equilibrium modeling together with the physicochemical characterization and the known coordination chemistry of the functional groups present in the NH₂-MIL-53(Al)@CS-EDTA composite.

Based on the abundance of oxygen- and nitrogen-containing functional groups in NH₂-MIL-53(Al)@CS-EDTA, coordination interactions between the metal ions and the adsorbent are expected to contribute to the overall adsorption behavior. EDTA provides multiple donor atoms capable of forming coordination complexes with heavy metal ions, while the NH₂-MIL-53(Al) framework facilitates accessibility to these functional groups. Consequently, the adsorption performance is plausibly associated with the synergistic contribution of the MOF framework and the chelating functionality of EDTA. However, direct confirmation of the dominant adsorption mechanism would require complementary post-adsorption characterization techniques. The presence of EDTA within the composite likely enhances selectivity and stability of metal ion binding via multidentate chelation, forming five- or six-membered ring structures with transition metals. In parallel, the MOF structure (NH₂-MIL-53(Al)) facilitates diffusion and accessibility to binding sites. The synergy between the MOF framework and the chelating functionality of EDTA results in a hybrid adsorbent that offers both high capacity and selectivity. Overall, the equilibrium adsorption data are more consistent with the Langmuir model under the investigated experimental conditions. Nevertheless, the isotherm analysis alone cannot conclusively establish monolayer adsorption or homogeneous adsorption sites. Accordingly, the adsorption mechanism is interpreted by combining the equilibrium results with physicochemical characterization and the known coordination behavior of the functional groups present in the composite, while recognizing that additional post-adsorption characterization would be required for direct mechanistic verification. Since the adsorption experiments were performed at a single temperature, thermodynamic parameters were not calculated in this study; future work should include multi-temperature adsorption experiments to evaluate ΔG°, ΔH°, and ΔS°.

**Table 3:** Parameters of Langmuir and Freundlich isotherm models for Cu²⁺, Fe²⁺, and Ni²⁺ adsorption onto NH₂-MIL-53(Al)@CS-EDTA, indicating adsorption capacities and model fitting coefficients.

Metal ion	Langmuir model			Freundlich model		
	q_max_, mg g^− 1^	K_L_, L mg^− 1^	*R* ^2^	K_F_, mg g^− 1^	*N*	*R* ^2^
Cu^2+^ Fe^3+^ Ni^2+^	31.8 28.1 21	1.45 0.046 0.54	0.9895 0.9831 0.9852	16.71 12.3 1.92	4.91 4.39 1.86	0.6606 0.9817 0.9738

### Previous literatures: maximum adsorption capacity of different adsorbents

To assess the practical applicability of NH₂-MIL-53(Al)@CS-EDTA in environmental remediation, particularly for the removal of heavy metals from aqueous media, it is essential to benchmark its adsorption performance against other reported adsorbents. The maximum adsorption capacity (q _max_) is commonly used as a key parameter in such comparisons. However, q max alone can be misleading, as it is highly dependent on specific experimental conditions—particularly the initial metal ion concentration and the adsorbent dosage. Elevated q max values may result from higher initial concentrations, which increase the driving force for mass transfer, or from lower adsorbent dosages, which enhance the calculated capacity due to fewer available binding sites.

Table 4 presents a comparison of q max values for NH₂-MIL-53(Al)@CS-EDTA (31.8 mg·g^−1^ for Cu²⁺, 28.1 mg·g^−1^ for Fe²⁺, and 21 mg·g^−1^ for Ni²⁺) with those of other adsorbents reported in the literature. While some materials—such as Fe₃O₄-ATP/EDTA/CS (267.94 mg·g^−1^ for Cu²⁺, 220.31 mg·g^−1^ for Ni²⁺) and polyaniline/calcium alginate composites (79.0 mg·g^−1^ for Cu²⁺)—demonstrate higher q_max_ values, these results were often obtained under experimental conditions that may not reflect real-world wastewater scenarios. Differences in pH, contact time, initial concentration, and adsorbent dosage significantly influence the reported capacities, making direct comparisons potentially misleading without a standardized testing framework.

The comparison in Table 4 shows that the maximum adsorption capacities of NH₂-MIL-53(Al)@CS-EDTA are moderate compared with some previously reported high-capacity adsorbents. Therefore, the practical value of the present composite should not be evaluated solely on the basis of qmax. Adsorption performance is strongly affected by experimental conditions, including initial concentration, adsorbent dosage, pH, contact time, temperature, and the nature of the tested system; therefore, direct comparison between different adsorbents should be interpreted cautiously.

Although some reported materials exhibit higher adsorption capacities, the NH₂-MIL-53(Al)@CS-EDTA composite offers a balanced performance profile by combining a relatively simple preparation route, biodegradable chitosan, EDTA-derived chelating functionality, the structural contribution of NH₂-MIL-53(Al), and reusability over five adsorption–desorption cycles. Thus, the main practical advantage of this material lies not in achieving the highest adsorption capacity, but in providing a multifunctional and environmentally compatible hybrid adsorbent capable of removing different metal ions under mild laboratory conditions. Further optimization of the composite composition, MOF loading, and operating conditions may improve its adsorption capacity in future studies.

**Table 4:** Comparison of the adsorption of studied NH_2_-MIL-53 (Al) @CS-EDTA with those in literatures.

Sorbent	Metal ions	Q_max_ (mg/g)	Operating conditions	BET surface area (m² g^–1^)	Ref.
Polyaniline/calcium alginate composite	Pb^2+^, Cu^2+^	357.0 (Pb^2+^), 79.0 (Cu^2+^)	pH 5.0; 0.01 g adsorbent/50 mL solution; C₀ = 5–40 mg L^−1^ for Cu²⁺ and 5–100 mg L^−1^ for Pb²⁺; contact time = 2 h; room temperature	N.R.	^8^
γ-Fe_2_O_3_ nanoparticles	Pb^2+^, Cu^2+^	69.0 (Pb^2+^), 34.0 (Cu^2+^)	pH 5.0; C₀ = 1–20 mg L^−1^; contact time = 3 h; temperature = 45 °C for Pb²⁺ and 25 °C for Cu²⁺	79.35	^10^
Amino functionalized mesoporous silica	Pb^2+^, Ni^2+^, Cd^2+^	57.7 (Pb^2+^), 12.4(Ni^2+^), 18.3(Cd^2+^)	pH 5.0; 0.5 g adsorbent/100 mL solution; C₀ = 10–70 mg L^−1^; contact time = 120 min; room temperature	N.R.	^20^
Fe_3_O_4_@CS−EDTA	Hg^2+^, Cd^2+^, Ni^2+^	232.70 (Hg^2+^), 121.40(Cd^2+^), 56.50 (Ni^2+^)	pH ≥ 4.5; contact time 30–120 min; room temperature; other conditions N.R.	0.527	^45^
_Fe3O4_-ATP/EDTA/CS	(Pb^2+^) (Cu^2+^, (Ni^2+^)	368.32 (Pb^2+^), 267.94 (Cu^2+^), 220.31 (Ni^2+^)	pH 5; dosage 0.3 g L^−1^; contact time 4 h; temperature 25 °C	51.81	^46^
chitosan-MOF composite	(Cu^2+^),(Ni^2+^)	50.6 (Cu^2+^), 60 (Ni^2+^)	pH 5; initial concentration 25 mg L^−1^; dosage 0.25 g L^−1^; contact time 8 h; temperature 60 °C for Cu²⁺ and 20 °C for Ni²⁺	N.R	^47^
Cysteine–NH_2_–MIL-53(Al)	(Pb^2+^), (Ni^2+^)	38.01 (Pb^2+^), 12.11 (Ni^2+^)	pH 6; initial concentration up to 60 mg L^−1^; dosage 10 mg/50 mL; contact time 50 min for Pb²⁺ and 40 min for Ni²⁺; room temperature	198.55	^48^
NH_2_-MIL-53(Al) @CS-EDTA	(Cu^2+^, Ni^2+^, Fe^2+^)	31.8 (Cu²⁺), 28.1 (Fe²⁺), 21 (Ni²⁺)	pH 6; initial concentration 20–100 mg L^−1^; dosage 2.5 g L^−1^; contact time 1 h; temperature 298 ± 1 K	0.18	This study

*N.R*. not reported or not accessible from the available source.

### Proposed adsorption mechanism of heavy metals onto NH₂-MIL-53(Al)@CS-EDTA

**Fig. 15:**
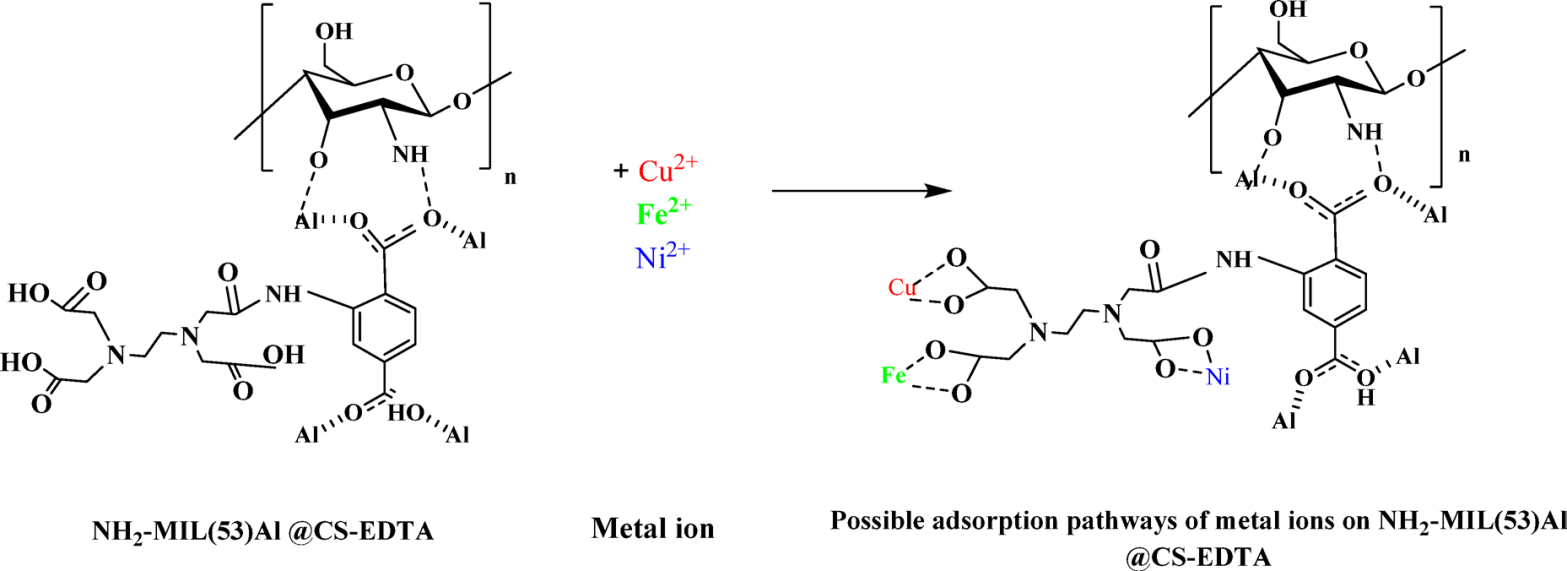
Proposed adsorption mechanism for the adsorption of Cu²⁺, Fe²⁺, and Ni²⁺ ions onto NH₂-MIL-53(Al)@CS-EDTA based on the characterization and adsorption results obtained in the present study.

The adsorption mechanism proposed for NH₂-MIL-53(Al)@CS-EDTA is inferred from the combined interpretation of physicochemical characterization (FTIR, XRD, SEM–EDX, BET, PZC), together with adsorption kinetics, equilibrium studies, and the known coordination chemistry of EDTA and chitosan. Since no post-adsorption spectroscopic analyses (e.g., FTIR, XPS, or zeta potential measurements) were performed, the following discussion should be regarded as a plausible mechanistic interpretation rather than direct experimental confirmation of individual adsorption pathways, as shown in Fig. 15.

The FTIR spectroscopy provide the successful functionalization and integration of the MOF, chitosan, and EDTA components. Characteristic peaks corresponding to –NH₂, –OH, and –COOH groups confirmed the presence of potential metal-binding functionalities within the composite structure. FTIR spectroscopy confirmed the successful incorporation of chitosan, EDTA, and NH₂-MIL-53(Al) within the hybrid composite through the presence of characteristic –NH₂, –OH, and –COOH functional groups. These groups are well known to possess strong coordination affinity toward transition-metal ions. Therefore, based on the observed adsorption behavior together with the known coordination chemistry of these functional groups, it is proposed that they participate in the adsorption process through metal complexation. However, because no spectroscopic characterization was performed after adsorption, the direct involvement of individual functional groups cannot be experimentally confirmed in the present study.

SEM observations revealed a rough and heterogeneous surface morphology with embedded MOF particles dispersed throughout the chitosan–EDTA matrix, while EDX analysis confirmed the expected elemental composition of the synthesized composite. These observations support the successful formation of the hybrid material and the availability of accessible surface functional groups. However, since SEM–EDX characterization was performed only before adsorption, these results should not be interpreted as direct evidence for the adsorption mechanism or the localization of adsorbed metal ions.

XRD analysis further corroborated the formation of a crystalline MOF structure, retained even after integration with chitosan and EDTA. The preservation of crystallinity implies that the structural integrity of the MOF was maintained, allowing its metal coordination sites (Al-O clusters) to remain available for interaction with target ions. The broad hump observed in the composite’s diffractogram additionally suggests partial amorphization due to polymer blending, which contributes to improved flexibility and enhanced interaction with aqueous media.

BET analysis showed that incorporation of NH₂-MIL-53(Al) modified the textural characteristics of the composite; however, the measured BET surface area remained relatively low compared with many pristine MOF-based adsorbents. Therefore, the adsorption performance observed in the present study is more appropriately attributed to the abundance of accessible oxygen- and nitrogen-containing functional groups provided by chitosan, EDTA, and the NH₂-MIL-53(Al) framework, which promote coordination and chelation interactions with metal ions. The BET results are thus interpreted as complementary structural information rather than direct evidence that physical porosity is the dominant factor governing adsorption.

The point of zero charge (PZC) of the NH₂-MIL-53(Al)@CS-EDTA composite was determined to be approximately pH 7.1. Accordingly, at pH values below this point, the surface carries a net positive charge, while it becomes negatively charged above pH 7.1. Under typical electrostatic considerations, metal cations such as Cu²⁺, Fe²⁺, and Ni²⁺ would be repelled at pH < 7.1 due to like-charge repulsion. However, the observed adsorption profile revealed that maximum uptake occurred near pH 6, which is below the PZC. This apparent contradiction can be explained by the predominance of chemical complexation mechanisms at this pH. The presence of EDTA and amino-rich chitosan provides multiple active functional groups—particularly carboxyl (–COOH) and amino (–NH₂) sites—that strongly chelate metal ions through coordinate bonding. These site-specific interactions effectively override electrostatic repulsion, allowing efficient metal ion adsorption even when the surface is positively charged. It is also worth noting that the pKa values of the carboxyl and amino groups (≈ 4.5–6.5) support their partial activation at pH 6, which facilitates coordination with metal ions without requiring full deprotonation. Therefore, the optimal adsorption at pH 6 results from a balance between reduced proton competition and the dominant chelation-driven binding mechanism.

The adsorption kinetics were better described by the pseudo-second-order model than by the pseudo-first-order model. This finding indicates that the adsorption process is consistent with a kinetic model frequently associated with surface interactions involving the available functional groups. However, kinetic fitting alone is insufficient to conclusively identify the adsorption mechanism or to confirm chemisorption without complementary spectroscopic evidence. Therefore, the kinetic results are interpreted only as being consistent with the proposed adsorption mechanism rather than definitive proof of chemical adsorption.

Equilibrium adsorption isotherm modeling showed a good correlation with the Langmuir model, suggesting predominantly monolayer adsorption onto relatively homogeneous active sites. The Langmuir-derived maximum adsorption capacities were 31.8 mg·g^−1^ for Cu²⁺, 28.1 mg·g^−1^ for Fe²⁺, and 21 mg·g^−1^ for Ni²⁺. This supports the hypothesis that specific binding sites on NH₂-MIL-53(Al) and EDTA chelation centers play dominant roles in metal uptake. The Freundlich model also showed high R² values, indicating heterogeneous surface interactions, particularly in the chitosan-EDTA domains, which offer multiple binding configurations.

Thermogravimetric analysis (TGA) demonstrated enhanced thermal stability of NH₂-MIL-53(Al)@CS-EDTA compared to individual components, suggesting strong intermolecular interactions within the composite. The reduced weight loss and increased residual mass confirm the formation of a stable network capable of resisting thermal degradation, further supporting its potential for real-world applications.

Based on the available experimental evidence, the adsorption process is most reasonably interpreted as being predominantly governed by coordination and chelation between the metal ions and the abundant oxygen- and nitrogen-containing functional groups present in the NH₂-MIL-53(Al)@CS-EDTA composite. Electrostatic interactions, depending on solution pH, may also contribute to the overall adsorption behavior. In contrast, ion exchange and pore filling cannot be experimentally distinguished in the present study and therefore are discussed only as possible secondary contributions rather than experimentally confirmed adsorption pathways.

This multistep mechanism is schematically illustrated in Fig. 14 and is validated by consistent trends across all experimental results. The combined evidence obtained from physicochemical characterization, pH-dependent adsorption behavior, kinetic modeling, and equilibrium isotherm analysis suggests that the adsorption of Cu²⁺, Ni²⁺, and Fe²⁺ onto NH₂-MIL-53(Al)@CS-EDTA is predominantly governed by the combined contribution of coordination interactions, chelation, electrostatic attraction, and other surface interactions associated with the chemically active functional groups of the composite. Since all adsorption experiments were conducted at a single temperature (298 ± 1 K), no thermodynamic parameters (ΔG°, ΔH°, or ΔS°) were determined. Consequently, no conclusions regarding the spontaneity, energetic favorability, or thermodynamic nature of the adsorption process are drawn in the present study. This highlights the adsorbent’s high selectivity, structural robustness, and environmental compatibility, positioning it as a viable candidate for advanced wastewater treatment applications.

The formation of NH₂-MIL-53(Al)@CS-EDTA involves both physical integration and covalent crosslinking processes. Initially, NH₂-MIL-53(Al) particles are homogeneously dispersed within the chitosan matrix through hydrogen bonding and electrostatic interactions between the MOF surface functional groups and the abundant –NH₂/–OH groups of chitosan.

Subsequently, EDTA dianhydride acts as a bifunctional crosslinker, reacting with the primary amine groups of chitosan via a nucleophilic acyl substitution mechanism. The –NH₂ groups attack the electrophilic carbonyl carbon of the anhydride ring, leading to ring opening and formation of stable amide bonds along with pendant carboxylic acid groups. Owing to the presence of two anhydride functionalities per EDTA molecule, inter-chain crosslinking occurs, generating a three-dimensional polymeric network.

Additionally, the residual carboxylate groups may coordinate with the Al³⁺ centers of NH₂-MIL-53(Al), further strengthening the interfacial interactions within the hybrid structure. Consequently, EDTA is incorporated into the composite as an immobilized structural component through covalent amide linkages rather than being present as a free chelating agent. This structural integration, together with the FTIR, XRD, and TGA characterization results, supports the formation of a stable hybrid network in which the chelating functionalities are retained within the composite architecture during adsorption. The developed structural design was therefore intended to maximize the accessibility of the active chelating groups while enhancing their stability under the investigated experimental conditions.

The chemical incorporation of EDTA through stable amide linkages, together with the FTIR and TGA characterization results, indicates that the chelating functionalities are integrated within the hybrid composite rather than being present as physically adsorbed species. This structural design is intended to enhance the stability of the active chelating groups during adsorption and regeneration under the investigated experimental conditions.

### Desorption and regeneration study

**Fig. 16:**
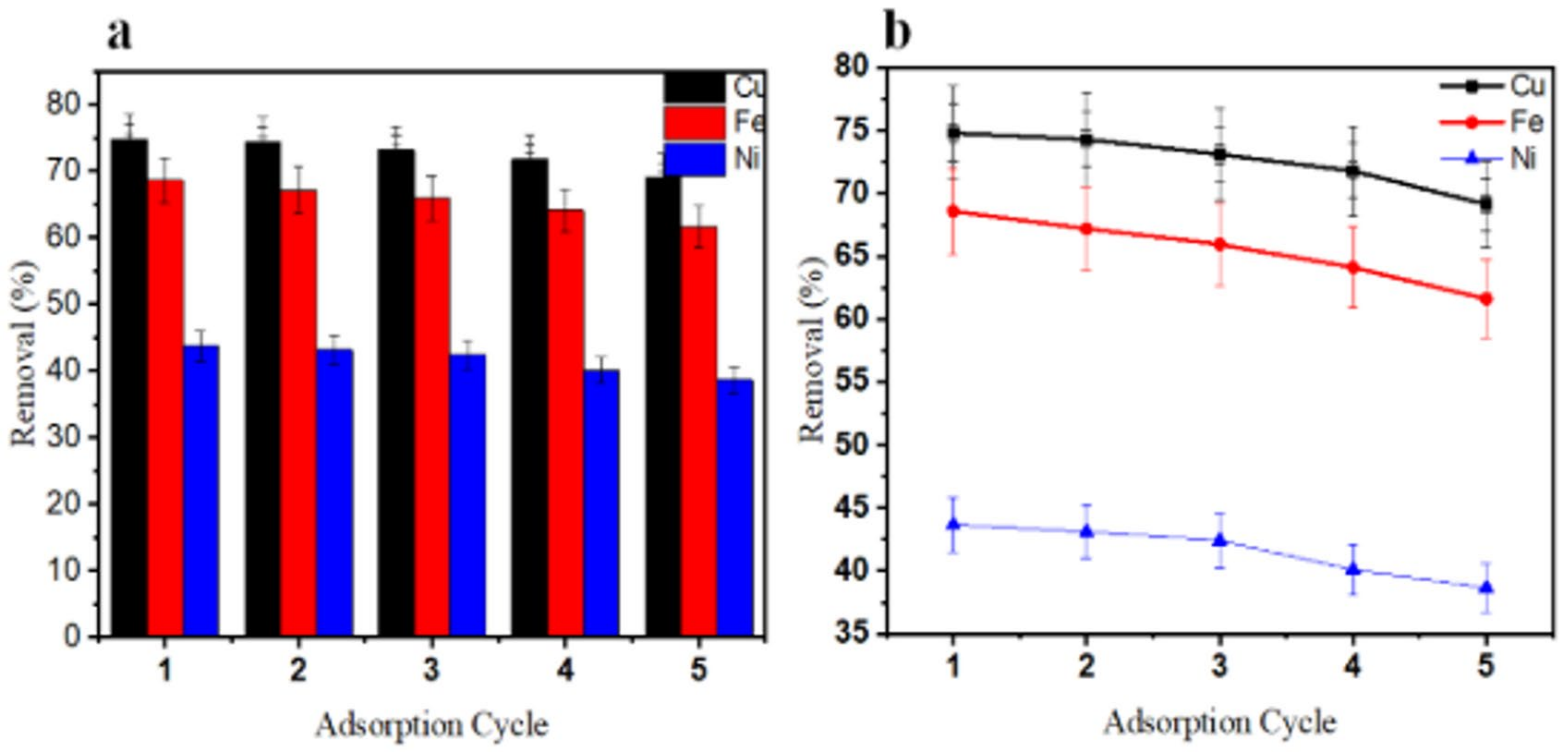
Performance of NH₂-MIL-53(Al)@CS-EDTA for adsorption and desorption over five regeneration cycles for Cu²⁺, Fe²⁺, and Ni²⁺ (Initial metal concentration = 100 mg·L^−1^, pH = 6, contact time = 1 h, adsorbent dose = 2.5 g L^−1^, temperature = 298.15 K, desorption agent: 2 M HNO₃).

In this study, the desorption of Cu²⁺, Fe²⁺, and Ni²⁺ ions were achieved using 2 M nitric acid. As shown in Table 5; Fig. 16, the composite retained a high level of adsorption performance over five cycles. The initial adsorption capacities for Cu²⁺, Fe²⁺, and Ni²⁺ were 29.5, 26.3, and 16.5 mg·g^−1^ respectively, which only slightly declined to 27.4, 24.4, and 15.2 mg·g^−1^ after the fifth regeneration cycle.

This moderate decline (~ 10%) indicates good operational durability and suggests that the composite maintained most of its adsorption functionality during repeated adsorption–desorption cycles. The regeneration results demonstrate that the developed composite retained its adsorption functionality over five consecutive adsorption–desorption cycles under the investigated experimental conditions. These findings support the functional reusability of the adsorbent within the examined operating window. The obtained regeneration results demonstrate the operational stability of the developed adsorbent throughout the investigated adsorption–desorption cycles, while avoiding extrapolation of these findings beyond the experimental conditions evaluated in the present study. The regeneration mechanism is likely based on proton exchange, where H⁺ ions from HNO₃ displace the adsorbed metal ions from the chelating functional groups such as –NH₂ and –COOH. These groups are restored to their original form after acid washing, ready for subsequent adsorption cycles.

Furthermore, the unmodified chitosan is known to degrade under strong acidic conditions such as 2 M HNO₃, as reported in previous studies^49,50^. However, functionalizing chitosan with EDTA and integrating it with a robust MOF (NH₂-MIL-53(Al)) significantly enhanced the acid resistance of the nanocomposite. This suggests the dual benefit of functionalization: enhancing adsorption efficiency and maintaining material stability during regeneration.

The ability to maintain high removal efficiency (> 60% for Cu²⁺ and Fe²⁺) even after multiple cycles positions NH₂-MIL-53(Al)@CS-EDTA as a promising and sustainable adsorbent for repeated use in heavy metal remediation from aqueous systems.

**Table 5:** Adsorption performance and removal efficiency of NH₂-MIL-53(Al)@CS-EDTA over five desorption–regeneration cycles for Cu²⁺, Fe²⁺, and Ni²⁺ ions.

Metals	Cu		Fe		Ni	
cycle number	Removal (%)	Adsorption capacity (mg g^− 1^)	Removal (%)	Adsorption capacity (mg g^− 1^)	Removal (%)	Adsorption capacity (mg g^− 1^)
1	74.80	29.57	68.59	26.31	43.70	16.48
2	74.29	29.48	67.18	25.8	43.12	16.56
3	73.10	28.6	65.95	25.88	42.42	16.8
4	71.78	29	64.10	25	40.10	15.24
5	69.12	27.4	61.61	24.4	38.66	15.15

### Limitations of the study

Despite the encouraging adsorption performance of the NH₂-MIL-53(Al)@CS-EDTA composite, several limitations of the present study should be acknowledged. First, the composite exhibited a relatively low BET surface area compared with many pristine MOF-based adsorbents, indicating that its adsorption performance is more likely governed by the abundance of accessible functional groups rather than by extensive physical porosity. In addition, the adsorption capacities obtained in this study are moderate compared with some highly engineered adsorbents reported in the literature. Future optimization of the composite formulation, MOF loading, and synthesis conditions may therefore be required to enhance adsorption capacity while maintaining the material’s environmental compatibility and regeneration ability. Second, the proposed adsorption mechanism is inferred from physicochemical characterization together with kinetic and equilibrium analyses; however, direct post-adsorption characterization techniques, such as FTIR, XPS, elemental mapping, or zeta-potential measurements, were not performed and would provide more definitive mechanistic evidence. Furthermore, adsorption experiments were conducted at a single temperature; therefore, thermodynamic parameters (ΔG°, ΔH°, and ΔS°) were not evaluated. The experiments were also carried out under batch conditions using synthetic aqueous solutions, which may not fully represent the complexity of real industrial wastewater containing multiple inorganic and organic constituents. Furthermore, the unexpected enhancement in adsorption observed in the presence of competing ions was not mechanistically investigated; therefore, additional multicomponent adsorption studies are recommended to clarify the origin of this behavior. In addition, the oxidation state of dissolved iron was not monitored during the adsorption experiments. Since the residual iron concentration was determined as total dissolved iron by atomic absorption spectroscopy (AAS), possible partial oxidation of Fe²⁺ to Fe³⁺ under aerobic conditions cannot be completely excluded. Future studies incorporating iron speciation analysis would provide a more comprehensive understanding of the adsorption behavior of individual iron species under the investigated condition. In addition, only five adsorption–desorption cycles were investigated; therefore, the long-term structural stability of the regenerated adsorbent remains uncertain. Moreover, no post-regeneration characterization (e.g., FT-IR, XRD, SEM, or BET analyses) was performed to evaluate possible structural changes after repeated use. Possible EDTA leaching during regeneration and the performance of the material under continuous-flow conditions also remain to be investigated. The batch adsorption results presented in this study provide a fundamental assessment of the adsorption behavior of the developed composite under controlled laboratory conditions. Building upon these findings, future investigations may evaluate the material under continuous-flow operation and realistic wastewater matrices to further assess its practical performance under dynamic treatment conditions.

## Conclusions

In this study, a novel NH₂-MIL-53(Al)@CS-EDTA hybrid nanocomposite adsorbent was successfully synthesized and characterized using FT-IR, SEM/EDX, XRD, BET, PZC, and TGA analyses. The developed material integrates the structural contribution of the NH₂-MIL-53(Al) framework with the chelating ability of EDTA and the functional properties of chitosan, resulting in a multifunctional hybrid adsorbent for heavy metal removal from aqueous media. Batch adsorption experiments demonstrated that the prepared composite exhibited moderate to good adsorption performance toward Cu²⁺, Fe²⁺, and Ni²⁺ ions, with optimum adsorption observed at pH 6 using an adsorbent dosage of 2.5 g L^−1^. The adsorption data were better described by the pseudo-second-order kinetic model and were more consistent with the Langmuir isotherm model under the investigated experimental conditions. However, these kinetic and equilibrium models alone are insufficient to conclusively identify the adsorption mechanism and should be interpreted together with the physicochemical characterization results. Based on the combined evidence obtained from material characterization and adsorption studies, the adsorption process is proposed to be predominantly governed by coordination and chelation between the heavy metal ions and the abundant oxygen- and nitrogen-containing functional groups present in the NH₂-MIL-53(Al)@CS-EDTA composite. Nevertheless, direct post-adsorption characterization techniques, such as FT-IR, XPS, elemental mapping, or zeta-potential analysis, were beyond the scope of the present study. Therefore, the proposed adsorption mechanism should be regarded as a plausible interpretation rather than direct experimental confirmation. Although the maximum adsorption capacities are moderate compared with some previously reported adsorbents, the developed composite offers a balanced combination of adsorption performance, structural stability, regeneration capability, and multifunctional surface chemistry. These characteristics, together with its effective performance toward different divalent metal ions under mild operating conditions, highlight its potential as a versatile adsorbent rather than one optimized solely for achieving the highest adsorption capacity. Future research should focus on evaluating the material under real wastewater conditions and continuous-flow systems, while also investigating its long-term structural stability, possible EDTA leaching during repeated use, and performance in more complex wastewater matrices containing dissolved salts, organic pollutants, antibiotics, and mixed contaminants. Furthermore, complementary post-adsorption spectroscopic characterization would provide more direct evidence of the adsorption mechanism and further strengthen the practical assessment of the developed composite.

## Data Availability

The datasets generated and/or analysed during the current study are available from the corresponding author on reasonable request.
